# Enhanced Selection of Assistance and Explosive Detection Dogs Using Cognitive Measures

**DOI:** 10.3389/fvets.2018.00236

**Published:** 2018-10-04

**Authors:** Evan L. MacLean, Brian Hare

**Affiliations:** ^1^School of Anthropology, University of Arizona, Tucson, AZ, United States; ^2^Department of Psychology, University of Arizona, Tucson, AZ, United States; ^3^Evolutionary Anthropology, Duke University, Durham, NC, United States; ^4^Center for Cognitive Neuroscience, Duke University, Durham, NC, United States

**Keywords:** cognition, assistance dog, detection dog, canine, behavior, cognition

## Abstract

Working dogs play a variety of important roles, ranging from assisting individuals with disabilities, to explosive and medical detection work. Despite widespread demand, only a subset of dogs bred and trained for these roles ultimately succeed, creating a need for objective measures that can predict working dog aptitude. Most previous research has focused on temperamental characteristics of successful dogs. However, working dogs also face diverse cognitive challenges both in training, and throughout their working lives. We conducted a series of studies investigating the relationships between individual differences in dog cognition, and success as an assistance or detection dog. Assistance dogs (*N* = 164) and detection dogs (*N* = 222) were tested in the Dog Cognition Test Battery, a 25-item instrument probing diverse aspects of dog cognition. Through exploratory analyses we identified a subset of tasks associated with success in each training program, and developed shorter test batteries including only these measures. We then used predictive modeling in a prospective study with an independent sample of assistance dogs (*N* = 180), and conducted a replication study with an independent sample of detection dogs (*N* = 90). In assistance dogs, models using data on individual differences in cognition predicted higher probabilities of success for dogs that ultimately succeeded in the program, than for those who did not. For the subset of dogs with predicted probabilities of success in the 4th quartile (highest predicted probability of success), model predictions were 86% accurate, on average. In both the exploratory and prospective studies, successful dogs were more likely to engage in eye contact with a human experimenter when faced with an unsolvable task, or when a joint social activity was disrupted. In detection dogs, we replicated our exploratory findings that the most successful dogs scored higher on measures of sensitivity to human communicative intentions, and two measures of short term memory. These findings suggest that that (1) individual differences in cognition contribute to variance in working dog success, and (2) that objective measures of dog cognition can be used to improve the processes through which working dogs are evaluated and selected.

## Introduction

Working dogs play a wide variety of important roles in human society, performing tasks ranging from assisting people with disabilities, to explosive and medical detection (1). Despite widespread demand, only a subset of dogs bred and trained for these roles are ultimately able to succeed as working dogs (2–4). Attrition from training programs, or failure to succeed after training, have important consequences with respect to public health (e.g., wait lists to receive certified assistance dogs) as well as the financial costs of breeding, training, and placing working dogs (e.g., through investment of resources in dogs that ultimately do not succeed). Therefore, there is an important need for objective measures that can predict whether individual dogs are likely to succeed in diverse types of working dog programs [reviewed in (5, 6)].

To date, most research on predictors of success as a working dog have focused largely on measures related to temperament and behavior. Studies of temperament have been motivated by the idea that working dogs are often utilized in highly stimulating environments, that these dogs frequently encounter unfamiliar people, other animals, and potentially startling stimuli, and that dogs must be able to remain calm, and task-focused in these situations. Similarly, inappropriate behaviors (e.g., excessive barking, scavenging, inappropriate elimination) can cause problems for dog handlers, or compromise a dog's ability to effectively perform his or her role. Studies across the last two decades have developed a wide range of approaches for assessing these characteristics, many of which serve as useful predictors of working dog success (2, 3, 7–17). For example, Wilsson and Sinn (16) found that scores on a principal component relating to a tendency to engage in tug-of-war, chasing, and interest in object retrieval, were positively associated with training success in the Swedish armed forces. In assistance dog populations, Duffy and Serpell (3) found that dogs prone to excitability, stranger- and dog-directed aggression, and social and nonsocial fear were less likely to successfully complete training. Lastly, Svobodová et al. (14) found that puppies that were more willing to chase and fetch a ball, and least reactive to noise, were the most likely to pass police dog certification. Therefore, previous studies have identified a range of temperamental and behavioral traits that relate to working dog training outcomes.

However, working dogs also face a variety of cognitive challenges, both in their initial training, and throughout their working lives (2, 18). Therefore, it is possible that individual differences in dog cognition also explain variance in aptitude for working roles (2). The cognitive skills that dogs require in these roles are likely to be diverse, extending beyond the basic learning mechanisms typically emphasized in dog training (e.g., operant and classical conditioning). For example, while animal trainers can shape behavior so that a dog associates the completion of a goal (e.g., retrieve the keys), with a social or food reward, trainers cannot train animals to flexibly and spontaneously respond to barriers that might prevent the completion of a trained goal (e.g., the closest door to the room where the keys are is closed, the keys are on the floor among many objects and are partially occluded by a book). In retrieving the keys, it is cognitive flexibility and not just temperament or trained responses that allows a dog to solve the problem. A dog's cognitive abilities allow her to mentally represent space and infer the need to take a detour (19, 20); to categorize objects as either being keys or not, and to inhibit bringing back incorrect object(s) (21–23); to maintain a mental representation of the referent of the verbal command (keys) in short-term memory even though it is not at first visible (24, 25); and to understand the communicative intention behind a human pointing gesture to infer the location of the occluded keys and finally retrieve them (26, 27).

Relative to studies of temperament, there have been very few investigations of whether individual differences in cognition relate to aptitude as a working dog. Bray et al. (2) recently tested young adult candidate guide dogs with a series of temperament and problem-solving tasks, and found that performance on a multistep problem-solving task was a significant predictor of subsequent success in the guide dog program. However, current work has employed relatively few cognitive measures, and has not explored associations between cognition and working dog outcomes in working roles beyond guide dogs.

The importance of assessing dog cognition broadly is evidenced through work describing the psychometric structure of individual differences in dog cognition. Specifically, individual differences in dogs are best described by multiple factors, reflecting psychological processes such as memory, understanding of communicative intentions, inhibitory control, and social engagement (28, 29). Thus, individual differences in dog cognition vary across multiple cognitive domains, yet we know little about which domains of cognition are most important for working dogs. Moreover, given that different working roles present different sets of job-specific challenges, it is probable that the aspects of cognition associated with working dog success will vary between different working roles. Therefore, the central challenges for this line of research are to (1) measure diverse cognitive processes when assessing links between cognition and working dog performance, and (2) identify the specific links between these aspects of cognition, and success in different working dog roles.

Here, we present a series of studies investigating associations between individual differences in dog cognition and success as an assistance or explosive detection dog. We implemented a similar research approach with both working dog populations. Within each population, we first conducted an exploratory study in which a sample of dogs was tested with a 25-item cognitive test battery, probing diverse aspects of dog cognition. We then identified associations between individual differences on items in this test battery, and measures of success as a working dog. Based on these preliminary findings, we then developed short-format test batteries including the subset of tasks that were most strongly associated with outcomes (specific to each population). Lastly, we implemented predictive models (Experiment 1) or a replication study with an independent sample of dogs (Experiment 2) to validate or confirm the associations between individual differences on the cognitive measures, and measures of success as a working dog.

## General methods (all populations)

During exploratory studies, large samples from both populations of working dogs were tested in the Dog Cognition Test Battery [DCTB; (28)]. The DCTB consists of 25 problem solving tasks designed to assess skills for reasoning about social and physical problems as well as domain-general cognitive processes. A detailed description of the DCTB, including its factor structure and implementation with the populations described here, is reported by MacLean et al. (28). All tasks in the DCTB are described briefly in Table 1, and were conducted by trained experimenters (university students and researchers). Detailed methods for these tasks are provided in the Supplemental Material. In all experiments, researchers were blinded to the training outcomes of dogs during testing, and dog trainers were blind to the results of the cognitive tests.

**Table 1:** Brief descriptions of measures included in the Dog Cognition Test Battery (DCTB).

**Task**	**Description**
Affect discrimination	Preference to approach unfamiliar human based on positive or negative affective cues
Arm pointing	Ability to use human arm pointing gesture to locate hidden reward
Causal reasoning	Use of visual and auditory cues to infer the location of a hidden reward
Contagious yawning	Tendency to yawn during auditory exposure to human yawning vs. control sounds
Cylinder	Ability to inhibit prepotent motor response in object retrieval task
Detour navigation	Navigation of shortest route around an obstacle
Gaze direction	Ability to use human gaze direction to locate hidden reward
Hiding-finding	Object permenence
Inferential reasoning	Ability to infer the location of a hidden reward through the principle of exclusion
Laterality: First step	Forelimb preference when initating a step off of a platform
Laterality: Object manipulation	Forepaw preference when physically manipulating an object
Marker cue	Ability to infer location of hidden reward when human uses a novel communicative marker
Memory - distraction	Memory for location of reward across delays while dog's attention is distracted
Odor control trials	Control trials ruling out ability to locate hidden food using olfaction
Odor discrimination	Discrimination and memory for which of two locations is baited using olfaction
Perspective-taking	Tendency to obey/disobey a command depending on whether human is watching
Reaching	Ability to infer reward location based on experimenter's reaching toward baited location
Retrieval	Tendency to retrieve object and return it to in front of experimenter
Reward preference	Preference for food or toy reward
Rotation	Egocentric vs. allocentric use of spatial cues
Sensory bias	Prioritization of visual vs. olfactory information when senses pitted against one another
Social referencing	Tendency to look at human face when joint social activity is interupted
Spatial perseveration	Ability to inhibit previously established motor pattern when environment changes
Spatial transpositions	Ability to track location of hidden reward across spatial transformations
Transparent obstacle	Ability to inhibit direct approach to experimenter when a detour is required
Unsolvable task	Help seeking from human vs. independent behavior when facing unsolvable task
Visual discrimination	Ability to learn arbitrary visual discrimination predicting reward location
Working memory	Memory for location of reward across temporal delays

*Detailed methods for all tasks are provided in MacLean et al. (28) and the Supplemental Materials*.

All testing was voluntary, and dogs were free to stop participating at any time. Subjects participated for food and toy rewards, and were not deprived of food or water. All experimental procedures were approved by the Duke University Institutional Animal Care and Use Committee (protocol number: A138-11-06). Inter-rater reliability was assessed for a randomly selected sample of 20% of the data and was excellent for all measures [mean ± SEM: kappa = 0.96; correlation = 0.94 ± 0.02; (28)].

## Experiment 1

### Exploratory study

#### Methods

##### Subjects

Candidate assistance dogs were tested at Canine Companions for Independence (CCI) in Santa Rosa, CA (*N* = 164; 107 females, 57 males, 19 Labrador retrievers, 4 golden retrievers, 141 Labrador retriever x Golden retriever crosses). CCI raises and places assistance dogs for diverse roles, including service dogs (placed with adults with physical disabilities), hearing dogs (placed with adults who are deaf or hard of hearing), skilled companion and facility dogs (placed with an adult or child with a disability under the guidance of a facilitator, or partnered with a facilitator in a health care, visitation, or education setting). Because hearing dogs are selected for a different behavioral phenotype than the other roles, hearing dogs (*N* = 21) were excluded from analysis. Dogs who aborted more than 2 tasks in the battery (*N* = 24) or were released for medical reasons (*N* = 8), were also excluded from analysis, yielding a final sample of 111 dogs included for exploratory analysis (mean age = 1.98 years, SD = 0.19 years).

##### Assistance dog outcome measures

After entering professional training and passing medical clearances, dogs in CCI's program either graduate and are placed in one of the roles described above, or are released for behavioral problems during training (a decision made by professional trainers without input from the researchers or knowledge of performance on cognitive tests). Therefore, success was coded as a categorical variable with two levels (graduate, release). In the sample for the exploratory study, 76 dogs graduated the program and 35 dogs were released. Assistance dogs were tested in the DCTB prior to obtaining an outcome in the program.

##### Analysis

For exploratory analysis, we implemented a variety of predictive modeling strategies. The cognitive data were prepared for analysis using a Box-Cox transformation (30) with missing data imputed using a K nearest neighbors approach. Exploratory analysis was conducted using eight different predictive modeling techniques to assess the utility of diverse modeling strategies, as well as consensus across models regarding variable importance. Specifically, we employed the following models from the *caret* package (31) in the R programming environment (32): (1) generalized linear model [GLM], (2) linear discriminant analysis [LDA], (3) regularized regression [RR], (4) partial least squares [PLS], (5) naïve Bayes classification [NB], (6) multivariate adaptive regression splines [MARS], (7) K nearest neighbors, and (8) random forest.

Because our aim was to develop a short-format battery using only the cognitive measures most strongly associated with training outcomes, we investigated the relative importance of predictor variables across models. To do so, we extracted the variable importance statistic from each of the training models, which reflects the relative importance of variables in a model (31), with values scaled to a range between 0 (unimportant) and 100 (most important). We also conducted univariate analyses (logistic regression) using each individual cognitive task as a predictor of training outcomes. We ranked the results of these analyses by *p*-value, and interpreted associations with the smallest *p*-values as those warranting future investigation. Across these analyses we identified 5 cognitive measures which were implicated as being strongly associated with training outcomes in exploratory analyses (causal reasoning [visual], spatial transpositions, inferential reasoning, cylinder, and social referencing). We further identified an additional 6 measures with more modest associations with training outcomes, or which were important covariates, that warranted further investigation (unsolvable task, odor discrimination, laterality: object manipulation, laterality: first step, arm pointing, and reward preference).

We then used data from these 11 measures to develop statistical models predicting training outcomes. Models were trained and evaluated using 4-fold cross validation, repeated 100 times (data randomly divided into 4-folds, 3 folds used for model construction, 1 fold used to assess model accuracy, with this process repeated 100 times). To assess model performance we used the cross-validated accuracy and area under the curve (AUC) from the receiver operating characteristic (ROC), a measure of sensitivity and specificity for a binary classifier. AUC values range between 0.5 and 1, with a value of 0.5 indicating a non-informative model, and a value of 1 indicating a perfectly predictive model.

Categorical predictions (graduate, release) were made using a probability threshold of 0.5 (i.e., predict release when predicted probability of graduation <0.5; predict graduate when predicted probability of graduation >0.5) but we retained predicted probabilities for additional analyses. Specifically, to assess whether accuracy was higher for observations with the highest and lowest predicted probability of success, we calculated cross-validated model accuracies for dogs with predicted probabilities of success in the 1st and 4th quartiles (at each iteration of the cross validation procedure).

## Results and discussion

The results from predictive models using the 11 candidate cognitive measures in the exploratory study are shown in Table 2. The best performing models (partial least squares, k nearest neighbors [*n* = 7]) yielded cross-validated AUCs of 0.76, and an overall cross-validated accuracy of 72%. However, all models tended to be much more accurate for dogs predicted to have the highest probabilities of graduating (Table 2). Specifically, for dogs in the 4th quartile of predicted probability of success (i.e., the 25% of dogs with the highest predicted probability of success), predictions were 87% accurate during cross validation. In contrast, for dogs in the 1st quartile of predicted probability of success, predictions tended to be less accurate (mean = 50%). Thus, we expected that future predictions would be most reliable for dogs with the highest predicted probabilities of success.

**Table 2:** Model statistics from the exploratory phase of Experiment 1.

	**LDA**	**GLM**	**RR**	**PLS**	**NB**	**MARS**	**KNN**	**RF**
Accuracy (training data)	0.79	0.85	0.82	0.79	0.85	0.70	0.79	1.00
AUC (training data)	0.86	0.90	0.89	0.86	0.92	0.62	0.86	1.00
Accuracy (CV)	0.68	0.70	0.71	0.72	0.69	0.64	0.72	0.69
AUC (CV)	0.71	0.74	0.75	0.76	0.68	0.55	0.76	0.70
1st quartile accuracy (CV)	0.88	0.91	0.90	0.91	0.82	0.73	0.87	0.90
4th quartile accuracy (CV)	0.51	0.54	0.55	0.56	0.52	0.40	0.46	0.45

*Accuracy (training data) and AUC (training data) reflect model performance with the training dataset. Metrics denoted as (CV) derive from cross validation with the training dataset (4-fold, 100 repeats). Columns represent the 8 different modeling strategies employed in this study. AUC, Area under the receiver operating characteristic curve; LDA, linear discriminant analysis; GLM, generalized linear model; RR, regularized regression; PLS, partial least squares; NB, naïve Bayes; MARS, multivariate adaptive regression splines; KNN, K nearest neighbors; RF, random forest*.

On a descriptive level, the largest differences between graduate and released dogs in the exploratory study were for the following tasks: spatial transposition, odor discrimination, causal reasoning (visual), unsolvable task (look at experimenter), inferential reasoning, social referencing. Specifically, graduate dogs scored higher than released dogs on the odor discrimination and inferential reasoning tasks, and made more eye contact with the experimenter during the social referencing and unsolvable tasks. However, release dogs scored higher than graduate dogs on the spatial transpositions task (one-cross condition) and the causal reasoning (visual) task. Therefore there was no clear pattern of graduates or releases systematically scoring higher across diverse cognitive measures.

### Prediction study

Following the exploratory study, we designed a shorter test battery consisting of the 11 tasks determined to be potentially promising measures during initial predictive modeling. Because some of these tasks included relatively few trials in their initial format, we added additional trials to assess whether more data from these measures would improve predictive power. Specifically, we implemented changes in the number of trials as follows: causal reasoning [visual]: 4 trials → 8 trials; spatial transpositions [one-cross condition]: 4 trials → 8 trials; inferential reasoning: 6 trials → 10 trials; odor discrimination: 6 trials → 10 trials. The revised test battery consisted of two test sessions (conducted on two consecutive days).

#### Subjects

We tested an independent sample of 180 dogs (i.e., none had participated in the exploratory study) in the revised assistance dog battery (115 females, 65 males, 43 Labrador retrievers, 4 golden retrievers, 133 Labrador retriever X golden retriever crosses). Of these, 33 dogs were excluded from analysis because they were transitioned into the hearing dog program (*N* = 19), released for medical reasons (*N* = 5), placed in a new program not represented in the training data (*N* = 7), or were still in training at the time of the analysis (*N* = 2). Twenty-six additional dogs were excluded from analysis due to missing data on more than two of the cognitive predictor variables. Therefore, our final sample for predictive modeling included 121 dogs (mean age = 1.88 years, SD = 0.23 years).

#### Procedure

Testing procedures were identical to those in the exploratory study with the exception that the revised battery included a smaller number of tasks, as well as additional test trials for some tasks, as described above. The order of tasks in the revised test battery for assistance dogs was: Day 1: warm-ups > causal reasoning (visual) > spatial transpositions > inferential reasoning > cylinder > mutual gaze; Day 2: warm-ups > unsolvable task > odor discrimination > laterality (object manipulation) > arm pointing > laterality (first step) > reward preference. Inter-rater reliability was assessed for ~40% of trials in the prediction study (Cohen's κ for discrete measures, Pearson correlation for continuous measures), and was excellent across measures (mean Cohen's κ = 0.98; mean Pearson's R: 0.96).

#### Analysis

To assess predictive validity, we used the predictive models in the exploratory study to predict training outcomes for dogs in the independent sample tested on the short-format battery. As in the exploratory study, we assessed model performance via accuracy and area under the curve (AUC) from the receiver operating characteristic (ROC). To assess the effect of including additional test trials in the short-format battery, we initially ran all predictive models both including and excluding data from these additional trials. Model performance was better using data including the additional trials, and we report these analyses below. Based on the results of the exploratory study, we expected that models would be most accurate for the subset of dogs with the highest predicted probability of success. To evaluate this prediction, we calculated accuracy separately for dogs with predicted probabilities of success in the 4th quartile of predicted probabilities.

## Results and discussion

At a descriptive level, all but one model (Naïve Bayes Classifier), predicted higher average probabilities of success for dogs that ultimately did graduate from the program, than for dogs who were released from training. However, one-tailed *t*-tests indicated that only the random forest model yielded predicted probabilities of success that were significantly higher for graduate than release dogs (Table 3). The best performing models (generalized linear model, random forest, and regularized regression) yielded AUCs of 0.60-0.61 (accuracy range: 68–74%; Table 4). Thus, overall model performance was considerably poorer than expected based on initial cross-validation with the training data set. However, the distribution of training outcomes varied considerably between the exploratory and prediction datasets, an issue that can seriously affect model performance (33). Specifically, in the exploratory dataset, 68% of dogs graduated from the program whereas considerably more dogs did so in the independent sample (77%). In addition, as expected based on the exploratory study, predictions were much more accurate for dogs in the 4th quartile of predicted probabilities of success. On average (across models), outcome predictions for dogs with predicted probabilities of success in the 4th quartile were 86% accurate (Table 4). Two models (linear discriminant analysis and random forest) yielded predictions that were 90% accurate for this subset of observations (Table 4).

**Table 3:** Results from *t*-tests comparing the predicted probability of success for dogs that were ultimately successful (graduates) or unsuccessful (releases) in the assistance dog training program.

	**All data**			**1st vs. 4th quartiles**		
	***t***	***df***	***p***	***t***	***df***	***p***
Generalized linear model	−1.49	43.35	0.07	−2.00	27.44	0.03
K nearest neighbors	−0.91	44.99	0.18	−0.96	13.62	0.18
Linear discriminant analysis	−1.15	41.45	0.13	−2.02	13.95	0.03
Multivariate adaptive regression splines	−1.38	37.71	0.09	−1.68	22.20	0.05
Naive bayes classifier	0.39	53.56	0.65	−0.24	15.99	0.41
Partial least squares	−1.10	50.18	0.14	−0.67	17.73	0.25
Random forest	−1.89	52.44	0.03	−1.88	22.53	0.04
Regularized regression	−1.42	45.85	0.08	−1.65	36.46	0.05

*First vs. fourth quartiles reflects this comparison restricting the data to dogs with predicted probabilities of success in the 1st and 4th quartiles. All tests were one-tailed to evaluate the directional hypothesis that predicted probability of success would be higher for graduate than release dogs*.

**Table 4:** Model statistics from the prediction study of Experiment 1.

	**LDA**	**GLM**	**RR**	**PLS**	**NB**	**MARS**	**KNN**	**RF**
AUC	0.57	0.61	0.60	0.57	0.52	0.57	0.55	0.60
Accuracy	0.64	0.68	0.70	0.67	0.71	0.76	0.71	0.74
Accuracy (upper quartile)	0.90	0.87	0.83	0.83	0.87	0.83	0.83	0.90

*AUC, Area under the receiver operating characteristic curve; LDA, linear discriminant analysis; GLM, generalized linear model; RR, regularized regression; PLS, partial least squares; NB, naïve Bayes; MARS, multivariate adaptive regression splines; KNN, K nearest neighbors; RF, random forest*.

**Figure 1:**
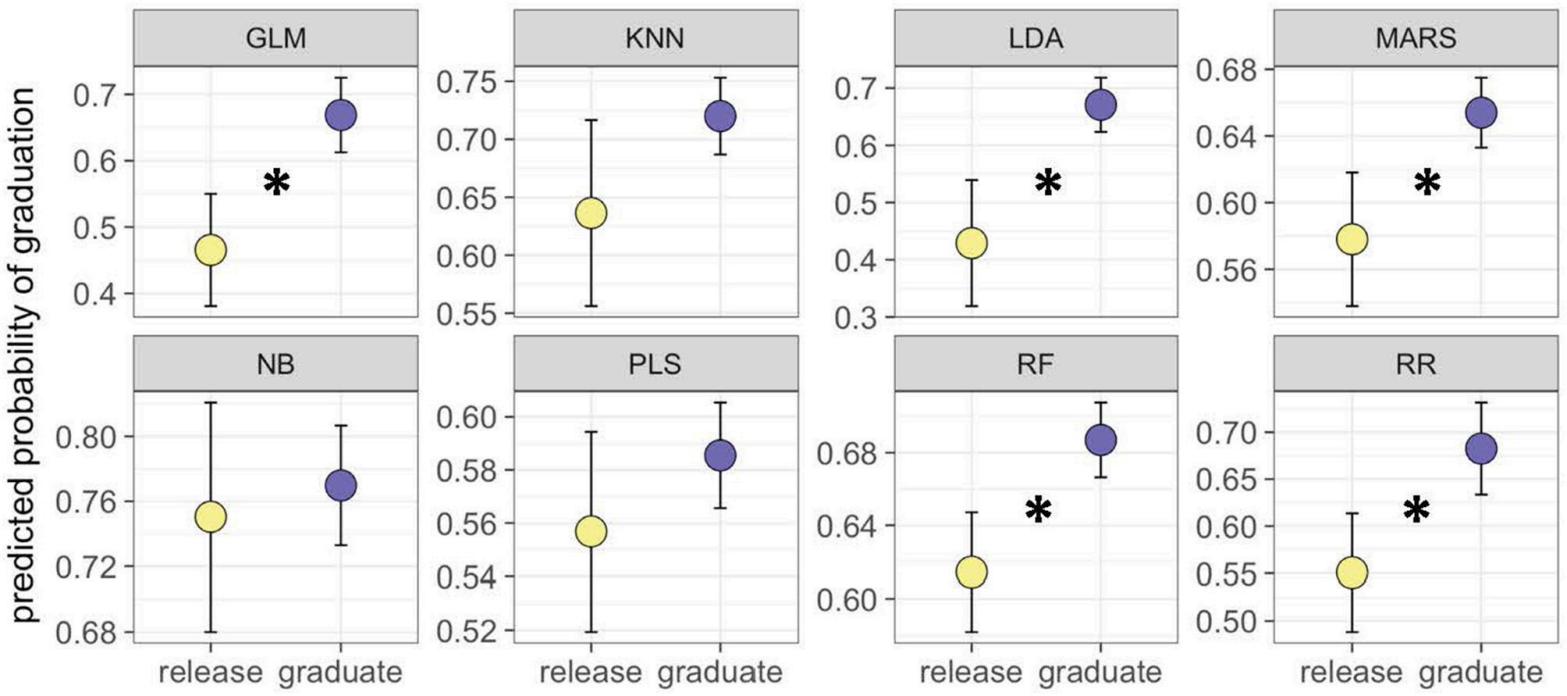
Mean predicted probability of success (±SEM) for dogs that ultimately graduated (blue points), or were released from the training program (yellow points), restricting data to dogs with predicted probabilities of the success in the 1st and 4th quartiles. Asterisks indicate significant differences at *p* < 0.05. LDA, Linear discriminant analysis; GLM, Generalized linear model; RR, Regularized regression; PLS, Partial least squares; NB, naïve Bayes; MARS, multivariate adaptive regression splines; KNN, K nearest neighbors; RF, random forest.

As a further test of the ability to discriminate between dogs most and least likely to succeed, we used one-tailed *t*-tests to compare the predicted probability of success for graduate and release dogs, restricting our analyses to dogs with predicted probabilities of success in the 1st and 4th quartiles (calculated separately for each model). In these analyses, 5 of 8 models produced significantly higher predicted probabilities of success for graduate compared to release dogs (Table 3; Figure 1). At a descriptive level, some differences in mean performance between graduate and release dogs were consistent between the exploratory and predictive studies, whereas others were not. Consistent with findings from the exploratory study, in the independent sample, graduate dogs again tended to make more eye contact with the experimenter in the unsolvable and social referencing tasks, and tended to score higher on the inferential reasoning task. However, in contrast to the exploratory study, graduate dogs scored higher on the spatial transpositions task, and scored lower on the odor discrimination task.

Overall, we were able to produce useful predictions regarding training outcomes with an independent sample, although relative to initial cross-validations, predictions for the independent sample tended to be less accurate. One important finding from this study was that predictions were much more accurate for the subset of dogs predicted to have the highest probability of success (with the strongest models performing at 90% accuracy in these cases). Therefore, from an applied perspective, we expect that it will be challenging to produce accurate predictions for all candidate assistance dogs, but that these measures and models may be particularly valuable for identifying the subset of dogs with the most potential for success. Given that these cognitive measures (1) can be collected in <2 h per dog, (2) do not require any training of dog participants, and (3) do not require specialized or costly equipment, these types of measures will provide a useful addition to the existing screening mechanisms employed by assistance dog agencies.

## Experiment 2

### Exploratory study

#### Methods

##### Subjects

Detection dogs were tested at K2 Solutions Inc. in Pinehurst, North Carolina. All detection dogs were Labrador retrievers (*N* = 222, 131 male, 91 female, mean age = 3.96 ± 1.66 years). Two-hundred and eight dogs completed the DCTB, and partial data were available for an additional 14 dogs.

##### Detection dog performance measures

Unlike the assistance dog organization, the detection dog provider did not employ a definitive metric to define success in the program. Therefore, we worked with the detection dog provider to assess diverse training and performance-related records which could be incorporated as proxies for success as a detection dog. These records included weekly training log entries, survey reports from trainers and individuals who had overseen a dog during deployment, standardized post-deployment evaluations, and dog status in the program. For several of these sources, we compiled information about 7 specific subcategories of dog performance, focusing on traits that program staff noted as important for detection work. These subcategories included the following: (1) Handling—ability to respond to directional signals when working off leash; (2) Temperament—nervous or fearful responses to loud noises or unfamiliar people and physical environments; (3) Motivation—eagerness to execute searches and follow verbal and gestural commands; (4) Handler dependence—overreliance on cues from the handler and limited ability to work independently; (5) Odor recognition—consistent detection of trained target odors; (6) Odor Response—appropriateness of behavioral response upon detection of a target odor; (7) False responses—tendency to indicate the presence of an odor when the odor was not present at that location. Below, we describe all data sources on dog performance and associated scoring protocols.

##### Training logs

Weekly training logs provided prose descriptions of dog behavior during training exercises (written by a dog trainer). Records primarily focused on aspects of performance needing additional attention (e.g., weaknesses rather than strengths). For each training session in the weekly log, researchers documented the occurrence of notes about weaknesses in the 7 subcategories described above. If no deficiencies were noted, the dog received a score of 0 for that category for a given week. If deficiencies were noted, they were assigned a prevalence score of 1–3, denoting the following categories: 1–rare and extremely minor weaknesses; 2–multiple, but sporadic weaknesses; 3–consistent patterns of deficiency. Most weekly logs contained notes about 3 or more days of training during the week. Logs in which 2 or fewer days of training were reported were excluded from analysis due to limited information for these periods. For each dog, we calculated a ratio of total scores in each behavioral category to the number of weekly training logs that were scored. Thus, higher ratios reflected more behavioral problems in training, controlling for the number of records available for analysis. Training log scoring was performed by two coders, with 20% of the sample coded by both individuals to assess inter-rater reliability (correlation). Reliability, was excellent for all measures (handling: R = 0.89; temperament: R = 0.93; motivation: R = 0.98; handler dependence: R = 0.96, odor recognition: R = 0.94; odor response: R = 0.97; false response: R = 0.99). Training log data for 162 dogs was available for analysis, with an average of 33 weeks of data per dog (SEM = 1.33 weeks).

##### Trainer surveys

For a subset of dogs in our sample (*N* = 34) we were able to administer quantitative surveys to the dog's primary trainer. Respondents rated dogs on a 3-point scale (above average, average, below average) relative to other dogs in the training program, with respect to each of the 7 behavioral subcategories described above.

##### Performance while deployed

For dogs that had previously deployed, we distributed a quantitative survey (identical to that used with trainers) to the individuals who were responsible for overseeing the dog during deployment. We obtained completed survey data for 62 dogs.

##### Post-deployment evaluation

Within 3 weeks of return from deployment, the provider performs a behavioral evaluation assessing temperament, detection and search abilities, obedience, and motivation. Each item on the evaluation is scored (by the provider) on a pass/fail basis. Within each category, we calculated the percent of passed items as the dependent measure. Evaluators also provided free-form comments on the dog's behavior at the time of the evaluation. Using these notes, coders assessed the presence/absence of deficiencies in the 7 behavioral subcategories described above. Data were available for 132 dogs. Coding of free-form comments was performed by two coders with 30% of the sample coded by both individuals to assess inter-rater reliability (Cohen's κ). Reliability was excellent for all measures (handling: κ = 1; temperament: κ = 0.88; motivation: κ = 0.84; handler dependence: κ = 1; odor recognition: κ = 0.93; odor response: κ = 0.78; false response κ = 0.94).

##### Status in program

The detection dog program assigned dogs a “status” relating to their fitness for future detection work. At the broadest level, dogs were considered serviceable if they were reserved for future use in the program, and unserviceable if they were being released from the program. Excluding dogs being released for non-behavioral reasons (e.g., medical problems), we used program status as a proxy measure for identifying the least and most successful dogs. Status records were available for 83 dogs that were identified as serviceable, or unserviceable due to behavioral reasons.

##### Analysis

Because detection dog performance could not be summarized using any single measure (the program did not use a definitive outcome), and data availability varied across measures, it was not feasible to build formal predictive models as in Experiment 1. Therefore, for the purpose of exploratory analysis, we conducted univariate analyses assessing associations between each performance measure described above, and individual item scores on the DCTB.

Scores on all outcome measures were discretized into two (training survey, post-deployment evaluation, program status, performance while deployed) or three (training log) quantile categories corresponding to dogs with below and above average scores on each measure (or below average, average, and above average for the measure discretized into 3 categories). For each performance measure, we conducted a *t*-test (2 category outcomes) or ANOVA (3 category outcomes) to test for differences on cognitive measures as a function of the discretized performance measure.

For exploratory analysis, we treated each analysis yielding a *p*-value < 0.05 as a significant association. Each significant association was then annotated to describe the direction of association between the cognitive and performance measure. For *t*-tests, these associations were either positive (higher scores on the cognitive measure associated with better performance) or negative (higher scores on the cognitive measure associated with worse performance). For the ANOVAs, we included a third category, “neutral” to annotate cases in which the omnibus test was significant, but there was no clear directional association with the performance measure (e.g., dogs in the above and below average categories performed similarly, whereas dogs in the average category deviated).

For aggregation across analyses, we assigned a score of −1 for each “negative” association, a score of 0 for a “neutral” association, and a score of +1 for each “positive” association. For each cognitive measure, we then added these scores (across analyses with the different performance measures) to derive an aggregate measure of the direction and strength of association between the cognitive and performance measures. For example, a cognitive measure that was significantly associated with 6 performance measures, with all 6 of these associations being positive (higher scores on the cognitive measure corresponding to better performance) would receive an aggregate score of 6. In contrast, a cognitive measure that was significantly associated with 6 performance measures, but with three of these associations being positive, and three being negative, would receive an aggregate score of 0 (−3 + 3 = 0). Thus, while we expected many false positives due to the large number of statistical tests, we predicted that the direction of false positive associations should be random. Consequently, we expected that the cognitive measures with the strongest positive or negative aggregate scores (consistent directional associations) would be those with the most robust and meaningful links to detection dog performance.

## Results and discussion

**Figure 2:**
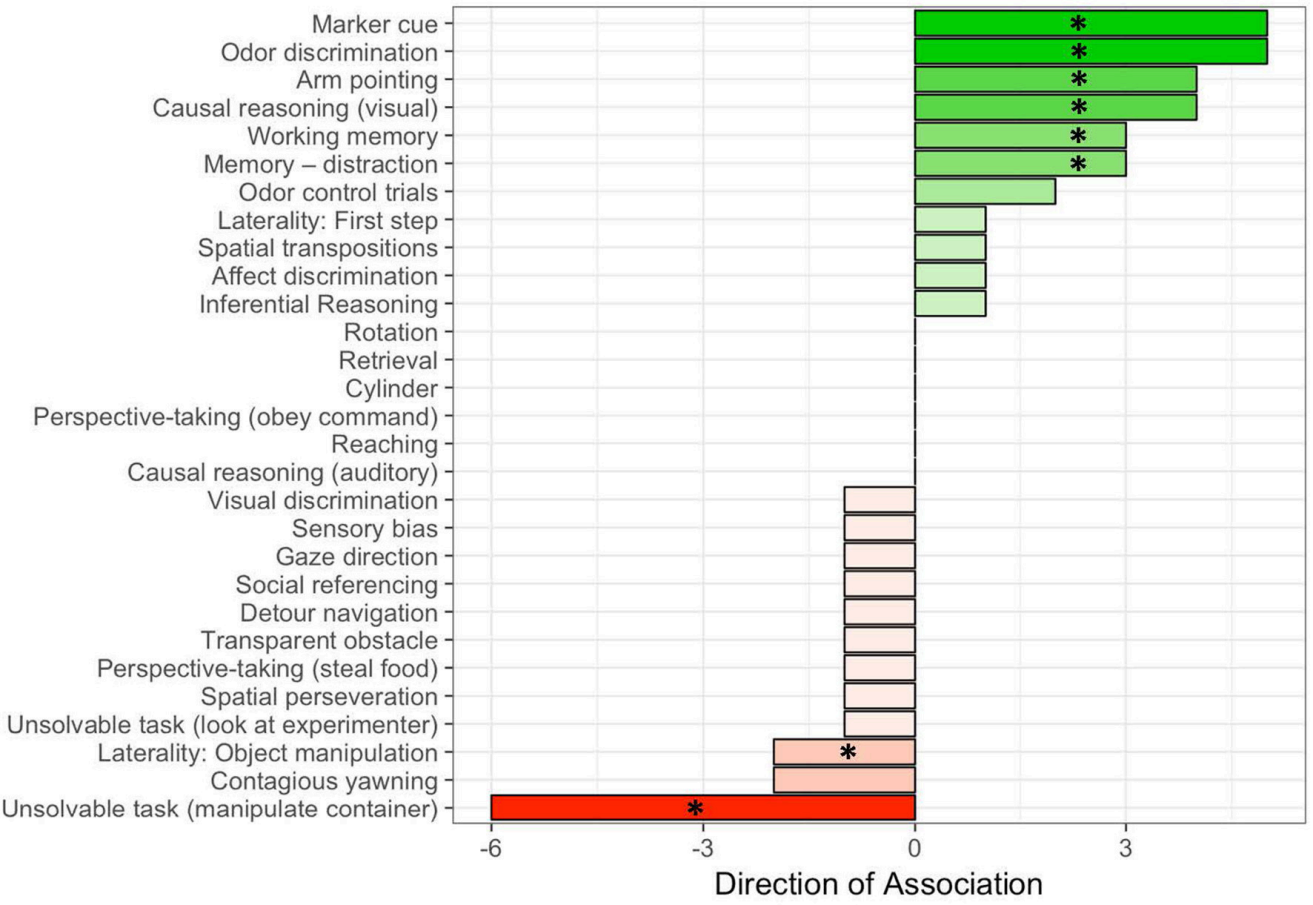
Aggregate scores describing the direction of the association between cognitive measures and metrics of success as a detection dog. Each significant positive association received a score of +1, and each significant negative association received a score of −1. The aggregate measure plotted on the x axis reflects the net of positive and negative association for each cognitive predictor in the test battery. Asterisks indicate tasks retained for the replication study.

Aggregate measures describing the association between cognitive tests and detection dog performance measures are shown in Table 5 and Figure 2. On average, there were 3.2 ± 0.4 associations with each cognitive measure. However, the mean aggregate score was 0.4 ± 0.4, which was not significantly different than the hypothesized value of 0, if false positives were equally likely to be positive or negative (one-sample *t*-test, *t*_28_ = 0.86, *p* = 0.40). However, the number of significant associations, and the directional consistency of these associations varied widely across cognitive measures.

**Table 5:** Distribution of positive, negative and neutral associations between cognitive measures and metrics of success as a detection dog from the exploratory study in Experiment 2.

**Measure**	**Associations**				**Aggregate score**
	**Total**	**Negative**	**Netural**	**Positive**	
Odor discrimination	5	0	0	5	5
Marker cue	5	0	0	5	5
Causal reasoning (visual)	4	0	0	4	4
Arm pointing	4	0	0	4	4
Memory—distraction	5	1	0	4	3
Working memory	5	1	0	4	3
Odor control trials	2	0	0	2	2
Inferential Reasoning	2	0	1	1	1
Affect discrimination	2	0	1	1	1
Spatial transpositions	3	0	2	1	1
Laterality: First step	1	0	0	1	1
Causal reasoning (auditory)	2	1	0	1	0
Reaching	2	1	0	1	0
Perspective-taking (obey command)	2	1	0	1	0
Cylinder	0	0	0	0	0
Retrieval	1	0	1	0	0
Rotation	2	1	0	1	0
Unsolvable task (look at experimenter)	3	2	0	1	−1
Spatial perseveration	5	3	0	2	−1
Perspective-taking (steal food)	1	1	0	0	−1
Transparent obstacle	10	5	1	4	−1
Detour navigation	1	1	0	0	−1
Social referencing	1	1	0	0	−1
Gaze direction	1	1	0	0	−1
Sensory bias	3	2	0	1	−1
Visual discrimination	6	3	1	2	−1
Contagious yawning	6	4	0	2	−2
Laterality: Object manipulation	2	2	0	0	−2
Unsolvable task (manipulate container)	6	6	0	0	−6

*Total indicates the total number of significant (p < 0.05) associations between each predictor variable and the outcome measures. Positive associations reflect cases in which higher scores on the cognitive measure were associated with better performance as a detection dog. Negative associations reflect cases where higher scores on the cognitive measure were associated with worse performance as a detection dog. Neutral associations indicate cases in which the test statistic was significant, but there was no clear directional association with the performance measure (e.g., dogs in the above and below average categories performed similarly, whereas dogs in the average category deviated)*.

Figure 2 depicts the aggregate score for each cognitive measure in the test battery. While some tasks (e.g., transparent obstacle) had many significant associations, the direction of these associations was highly variable, yielding an aggregate score near 0. In contrast, five cognitive measures had four or more significant associations, all of which were positive (i.e., higher scores on the cognitive measure linked to better measures of performance as a detection dog), and two additional measures had five significant associations, with 80% of these being positive. One cognitive measure had 6 associations with performance metrics, all of which were negative. Based on these results, and the aim of developing an approximately 1 h short-format test battery, we retained all measures with an aggregate score of ≥ |3| (marker cue, odor discrimination, arm pointing, causal reasoning [visual], working memory, memory—distraction, and unsolvable task). We opted to retain one additional measure which yielded two negative associations with performance metrics (laterality: object manipulation) due to the simplicity and potential utility of this measure.

The cognitive measures yielding consistent directional associations with detection dog performance included measures of sensitivity to human communication, short-term memory, odor discrimination, causal reasoning, and persistence at an unsolvable task. Several of these tasks index processes that are likely to be important for dogs performing off-leash explosive detection. For example, off-leash detection dogs are required to use gestural communication from a human handler when executing search routes, and individual differences in sensitivity to human communication may be an important determinant of success in this aspect of detection work. Similarly, detection dogs rely on short-term memory in a variety of situations ranging from memory for recent commands, to locations recently searched, and odorants (or the strength thereof) recently encountered. Lastly, detection dogs are required to make olfactory discriminations, and individual differences in spontaneous odor discrimination tasks may predict a dog's potential for employing these skills during trained detection work. Therefore, several of the positive associations from the exploratory study can be intuitively interpreted with respect to the requirements of detection work.

One limitation of this study was that because there was no definitive outcome measure in the detection dog population, it was not possible to develop formal predictive models as we did with the assistance dogs. Because the outcomes we recorded were not available for all dogs, and data availability varied widely between measures, it was similarly not possible to develop a unified outcome measure (e.g., through dimension reduction). However, by relying on a diverse set of outcome measures, it is possible that this type of analysis provides a more sensitive measure of working dog performance than a simple pass/fail type of metric.

## Replication study

To assess the replicability of associations from the exploratory study, we tested an independent sample of detection dogs in a short-format assessment consisting of the measures most strongly associated with detection dog performance in the exploratory study.

### Methods

#### Subjects

Ninety Labrador retriever dogs (60 male, 30 females) participated in the replication study. All dogs were from the detection dog population described above, and none of them had participated in the initial exploratory study.

#### Procedure

Testing procedures were identical to those in the exploratory study with the exception that a smaller number of tasks were employed, and tasks were implemented in a novel order. Unlike Experiment 1, we did not include additional test trials for any of the measures in this replication study. The order of tasks in the replication study was: warm-ups > arm pointing > marker cue > odor discrimination > working memory > memory—distraction > unsolvable task > causal reasoning (visual) > laterality: object manipulation. We assessed inter-rater reliability (Cohen's κ for discrete measures, Pearson correlation for continuous measures) for ~20% of all trials, and reliability was excellent across measures (mean Cohen's κ = 0.97; mean Pearson's R: 0.91).

#### Performance measures

As in the exploratory study, we obtained and scored records to be used as a proxy of success as a detection dog. Our primary performance measure was scoring of weekly training logs, as described above (*N* = 67 dogs). Two coders rated 20% of observations and inter-rater reliability was excellent for all measures (handling: R = 0.99; temperament: R = 1.0; motivation: R = 0.89; handler dependence: R = 0.99, odor recognition: R = 0.91; odor response: R = 0.97; false response: R = 0.99). For dogs in the replication study, the ratio scores (problems per category to weeks of data) were correlated with the number of weeks of data available. To control for this confound, we used linear models predicting the ratio score as a function of weeks of available data, and extracted residuals from these models as an adjusted measure of performance. Prior to analysis, residuals were multiplied by −1 so that higher values corresponded to better performance in the program.

For dogs in the replication study we also gained access to additional electronic records which described (trainer perceptions of) weekly performance for each dog using an ordinal scale (“excellent,” “good,” “fair,” “poor”). These electronic records were obtained for 71 dogs, with a mean of 97.4 records per dog (SEM = 8.9). To quantify these ordinal scores, for each dog we (a) calculated the percent of records achieving each of the different ordinal ratings, (b) multiplied each percentage by the following weightings: excellent = 1, good = 0.66, fair = 0.33, poor = 0, and (c) summed these values to obtain an overall numerical score. Thus, overall numerical scores were bounded from 0 (all ratings = poor) to 100 (all ratings = excellent). Observed overall scores had a mean of 59, and ranged between 24 and 70.

The other measures of dog performance originally used in the exploratory phase were unavailable for dogs in the replication study, and thus could not be included in analysis.

#### Analysis

To replicate the approach used in the exploratory study, we conducted univariate analyses predicting the performance outcome measures described above as a function of scores on each of the cognitive tasks. All statistical tests were run as linear models with the predictor and outcome variables converted to z-scores to facilitate interpretation of regression coefficients.

For each analysis we recorded the β coefficients describing the relationship between the cognitive predictor variable and the detection dog performance measure as an outcome. To summarize results from these analyses we (1) calculated the mean and standard error of the β coefficients for each predictor variable, and (2) performed a one-tailed, one-sample *t*-test on the distribution of these β coefficients for each predictor variable, testing the null hypothesis that the β coefficients would have a mean of 0. The direction of the alpha region for the one-tailed *t*-tests was assigned based on whether we hypothesized a positive or negative association with the cognitive predictor variable, based on the results of the exploratory study. Therefore, our main predictions were that cognitive measures that were positively associated with detection dog performance in the exploratory study would also have positive β coefficients in the replication study, and vice versa for associations determined to be negative in the exploratory study.

## Results

**Figure 3:**
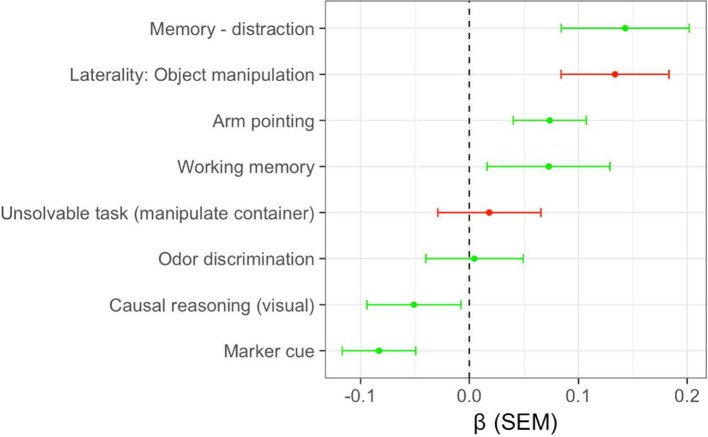
Mean and standard error of the standardized regression coefficients (ß) for cognitive measures in the replication study. Green points and error bars indicate measures which were positively associated with outcomes in the exploratory study. Red points and error bars indicate measures that were negatively associated with outcomes in the exploratory study.

The mean and standard error of the β coefficients associated with each cognitive predictor are shown in Figure 3. Four of the six measures which were positively associated with detection dog performance in the exploratory study, on average, also had positive β coefficients in the replication study. For two of these measures (memory—distraction, arm pointing) the distribution of β coefficients had a mean significantly >0 (Table 6), suggesting consistent positive associations with detection dog performance. However, two cognitive measures which were positively associated with performance in the exploratory study were negatively related to performance in the replication study (Figure 3; Table 6). In addition, both cognitive measures that were negatively associated with performance in the exploratory study had, on average, positive β coefficients in the replication study. Therefore, the replication study confirmed a subset of findings from the exploratory study, but did not replicate other findings.

**Table 6:** Mean and standard error of regression coefficients from the replication study.

**Predictor**	**Exploratory study**	**Replication study**				
	**Direction of association**	**Regression models**		**One-sample** ***t*****-test (**β **coefficients)**		
		**β (Mean)**	**β SEM**	***t***	***df***	***p***
Marker cue	Positive	−0.08	0.03	−2.46	7	0.98
Odor discrimination		0.00	0.04	0.10	7	0.46
Arm pointing		0.07	0.03	2.19	7	0.03
Causal reasoning (visual)		−0.05	0.04	−1.18	7	0.86
Working memory		0.07	0.06	1.29	7	0.12
Memory – distraction		0.14	0.06	2.43	7	0.02
Laterality: Object manipulation	Negative	0.13	0.05	2.69	7	0.98
Unsolvable task (manipulate container)		0.02	0.05	0.39	7	0.64

*β (mean) reflects the mean regression coefficient describing the relationship between a cognitive measure and the outcome variables (metrics of performance as a detection dog). T-test statistics correspond to one-sample t-tests comparing the distribution of β coefficients for each predictor to the null expectation (0)*.

As in the exploratory study, multiple measures of short-term memory were positively associated with detection dog performance. Similarly, individual differences in sensitivity to human gestures (arm pointing) was associated with better detection dog outcomes, in both the exploratory and replication phases. Although odor discrimination, causal reasoning (visual), and use of an arbitrary communicative marker were all positively associated with performance in the exploratory study, none of these tasks maintained strong associations across the replication. Additionally, the two tasks that were negatively associated with detection dog performance in the exploratory study (laterality: object manipulation, unsolvable task [look at experiment]) were unrelated to detection dog outcomes in the replication.

The use of an exploratory and confirmatory approach illustrates the importance of replication in developing predictive measures. Spurious or weak results are less likely to be upheld across analyses with independent datasets, whereas the most promising measures should yield comparable findings across multiple iterations of behavioral testing and analysis. In the current experiment, it is possible that some initial findings did not replicate because these associations were spurious or relatively weak. However, many of the outcome measures used in our exploratory study were not available for dogs in the replication study, which may also account for limited reproducibility in some cases.

In sum, these findings indicate that simple measures of short-term memory and sensitivity to human gestural communication are reliably associated with performance as a detection dog, and suggest that these measures may provide a simple and rapid approach for evaluating a dog's potential for this role. The current work identifies a subset of simple cognitive measures that can be easily incorporated into such a prospective study.

## General discussion

Across a series of studies with candidate assistance dogs and detection dogs, we assessed associations between individual differences in cognition, and success as a working dog. In both populations we initially used exploratory analyses with a large sample of dogs tested on a broad array of cognitive tasks. We then developed shorter test batteries comprised of only the items most strongly associated with outcomes within each population. Lastly, we collected data on these revised sets of measures with independent samples and used predictive models (assistance dogs) or a replication study (detection dogs) to assess the utility of these cognitive measures for predicting working dog outcomes. In both populations we identified cognitive measures associated with working dog success. In the assistance dog population, predictive models developed in the exploratory study were effective at prospectively predicting training outcomes in an independent sample, with model performance being best for dogs predicted to have the highest (vs. the lowest) probability of success. In the detection dog population, our replication study confirmed positive associations between individual differences in short-term memory, sensitivity to human gesture, and measures of success as a detection dog. Therefore, our findings suggest that measures of dog cognition provide a useful approach for predicting working dog aptitude, and support the hypothesis that individual differences in cognition may be an important determinant of success in these roles (2).

Importantly, the particular aspects of cognition associated with working dog success varied between the two study populations, consistent with the notion that different working roles may require different cognitive skillsets. In the assistance dog population, successful dogs were characterized by a greater tendency to engage in eye contact with a human when faced with an unsolvable task, or when a joint social activity was disrupted (social referencing), as well as higher scores on an inferential reasoning task. Given that assistance dogs work closely with a human partner, and must be highly responsive to this person, it is likely that a natural tendency to attend to the human's face, and seek information from this person, is fundamental to a dog's success in this role. In the detection dog population, we found the strongest associations with measures of short-term memory and sensitivity to human gestural communication. Given that these dogs work off-leash at a distance from a human handler, it is likely that the ability to use human gestural communication provides an important skillset for effective detection work. Similarly, because detection dogs must efficiently search complex physical environments, and maintain verbal commands in memory while executing searches, short-term memory is probably critical for several aspects of successful detection work.

Our findings support the hypothesis that different types of cognition have evolved in a variety of animals—including dogs (28, 29, 34, 35). In our previous study describing the psychometric structure of the DCTB (i.e., the same data used here), measures of sensitivity to communicative intentions, memory processes, and eye contact with humans, all loaded on different factors (28). Therefore, our current findings are consistent with the hypothesis that the cognitive skills linked to working dog success reflect processes in distinct cognitive domains, that can vary independently of one another. This provides evidence that individual variation across these different factors, or types of cognition, is also related to how dogs solve a variety of problems in the real world. In other words, these experimental measures have ecological validity (36). An individual's cognitive profile can increase his or her potential to either succeed or fail in performing trained behaviors effectively—with different profiles being predictive of success with different sets of problems (e.g., assisting people with disabilities vs. explosive detection). This also leads to the prediction that future studies with other working dog populations will identify other aspects of cognition that are important for other working roles. If correct, it is unlikely that a construct such as “general intelligence” will be sufficient for assessing (cognitive) aptitude in candidate working dogs. At a practical level, this suggests that there will not be a single (ideal) cognitive phenotype that can be selected or screened for across all working dog populations.

One important challenge in assessing cognitive predictors of working dog success will continue to be how success is defined and operationalized. In the assistance dog population, training success was independently defined by the dog provider, and operationalized as whether a dog graduated the program [a common metric of success for studies with assistance dogs; (2, 3, 7)]. Although clearly defined, and relevant to the practical challenges that motivate predictive modeling (e.g., identifying dogs most and least likely to complete training), the use of a dichotomous outcome may obscure meaningful differences between dogs within the successful and unsuccessful groups. In the detection dog population there was no single metric available to quantify success, and thus we relied on diverse approaches ranging from scoring training records to surveys with trainers and individuals overseeing dogs during deployment. These data sources likely reflect a large degree of subjectivity. Additionally, many of these data sources were not available for dogs in our study, yielding variance in statistical power across analyses, and precluding the development of a single composite metric of success. Therefore, in addition to continued research on the cognitive and behavioral traits that predict aptitude for working roles, there is also an important need for the development and validation of objective measures that can more robustly quantify success in these roles.

Despite these limitations, Our findings speak to the validity of spontaneous, non-verbal cognitive measures in capturing meaningful differences in real world problem solving behavior (34). They suggest that in dogs (1) individual differences in cognition contribute to variance in working dog success, and (2) that experimental measures of these individual differences can be used to improve the processes through which working dogs are evaluated and selected. Importantly, we expect that cognitive measures will be useful in addition to, rather than as an alternative to current methods of dog selection. A wide range of traits, including aspects of physical health, behavior, temperament, and cognition, make important contributions to working dog success. Thus, the development and validation of measures that probe this diverse range of phenotypic characteristics will be critical to enhancing working dog selection. Collectively, our findings contribute to a rapidly growing body of research on working dog selection, and suggest that embracing a broad view of the characteristics required of successful working dogs—including temperamental and cognitive traits, as well as the interactions between them (18, 37, 38)–will provide a powerful and integrative approach for future research.

## Ethics statement

This study was carried out in accordance with the recommendations of the Duke University IACUC, and was approved by the Duke University IACUC (protocol #: A138-11-06).

## Author contributions

EM and BH designed and conducted the research. EM analyzed the data. EM and BH wrote the paper.

### Conflict of interest statement

The authors declare that the research was conducted in the absence of any commercial or financial relationships that could be construed as a potential conflict of interest.

## References

[1] Wells DL. Domestic dogs and human health: an overview. Br. J. Health Psychol. (2007) 12:145–56. 10.1348/135910706X103284 17288671

[2] Bray EE Sammel MD Cheney DL Serpell JA Seyfarth RM. Effects of maternal investment, temperament, and cognition on guide dog success. Proc Natl Acad Sci. (2017) 114:9128–33. 10.1073/pnas.1704303114 28784785 PMC5576795

[3] Duffy DL Serpell JA Predictive validity of a method for evaluating temperament in young guide and service dogs. Appl Anim Behav Sci.(2012) 138:99–109. 10.1016/j.applanim.2012.02.011

[4] Harvey ND Craigon PJ Blythe SA England GC Asher L. An evidence-based decision assistance model for predicting training outcome in juvenile guide dogs. PLoS ONE(2017) 12:e0174261. 10.1371/journal.pone.0174261 28614347 PMC5470660

[5] Cobb M Branson N McGreevy P Lill A Bennett P. The advent of canine performance science: offering a sustainable future for working dogs. Behav. Processes (2015) 110:96–104. 10.1016/j.beproc.2014.10.012 25444772

[6] MacNamara M MacLean E Selecting animals for education environments. In: Gee NR, Fine AH, McCardle P, editors. How Animals Help Students Learn: Research and Practice for Educators and Mental-Health Professionals. New York, NY: Routledge (2017). p. 228.

[7] Batt LS Batt MS Baguley JA McGreevy PD The value of puppy raisers' assessments of potential guide Dogs' behavioral tendencies and ability to graduate. Anthrozoos (2009) 22:71–6. 10.2752/175303708X390482

[8] Berns GS Brooks AM Spivak M Levy K. Functional MRI in awake dogs predicts suitability for assistance work. Sci Rep. (2017) 7:43704. 10.1038/srep43704 28266550 PMC5339790

[9] Duffy DL. Bringing objectivity to working dog selection: the role of lateralization measures. Veter J. (2012) 192:262–3. 10.1016/j.tvjl.2011.12.012 22280880

[10] Goddard M Beilharz R Early prediction of adult behaviour in potential guide dogs. Appl Anim Behav Sci. (1986) 15:247–60. 10.1016/0168-1591(86)90095-X

[11] Jones AC Gosling SD Temperament and personality in dogs (Canis familiaris): a review and evaluation of past research. Appl Anim Behav Sci (2005) 95:1–53. 10.1016/j.applanim.2005.04.008

[12] Leotta R Voltini B Mele M Curadi MC Orlandi M Secchiari P Latent variable models on performance tests in guide dogs. 1. Factor analysis. Ital J Anim Sci. (2010) 5:377–86. 10.4081/ijas.2006.377

[13] Mizukoshi M Kondo M Nakamura T Evaluation of the potential suitability of guide dog candidates by continuous observation during training. J Veter Behav. (2008) 3:193–8. 10.1016/j.jveb.2008.05.002

[14] Svobodová I Vápeník P Pinc L Bartoš L Testing German shepherd puppies to assess their chances of certification. Appl Anim Behav Sci. (2008) 113:139–49. 10.1016/j.applanim.2007.09.010

[15] Weiss E. Selecting shelter dogs for service dog training. J Appl Anim Welfare Sci. (2002) 5:43–62. 10.1207/S15327604JAWS0501_4 12738588

[16] Wilsson E Sinn DL Are there differences between behavioral measurement methods? A comparison of the predictive validity of two ratings methods in a working dog program. Appl Anim Behav Sci. (2012) 141:158–72. 10.1016/j.applanim.2012.08.012

[17] Wilsson E Sundgren P-E The use of a behaviour test for selection of dogs for service and breeding. II Heritability for tested parameters and effect of selection based on service dog characteristics. Appl Anim Behav Sci. (1997) 54:235–41. 10.1016/S0168-1591(96)01175-6

[18] Bray EE Sammel MD Seyfarth RM Serpell JA Cheney DL. Temperament and problem solving in a population of adolescent guide dogs. Anim Cogn. (2017) 20:923–39. 10.1007/s10071-017-1112-8 28695349

[19] Fiset S Landry F Ouellette M. Egocentric search for disappearing objects in domestic dogs: evidence for a geometric hypothesis of direction. Anim Cogn. (2006) 9:1–12. 10.1007/s10071-005-0255-1 15750805

[20] Pongracz P Miklosi A Kubinyi E Gurobi K Topal J Csanyi V Social learning in dogs: the effect of a human demonstrator on the performance of dogs (Canis familiaris) in a detour task. Anim Behav. (2001) 62:1109–17. 10.1006/anbe.2001.1866

[21] Kaulfuß P Mills D. Neophilia in domestic dogs (Canis familiaris) and its implication for studies of dog cognition. Anim Cogn. (2008) 11:553–6. 10.1007/s10071-007-0128-x 18183436

[22] Pilley JW Reid AK. Border collie comprehends object names as verbal referents. Behav Processes (2011) 86:184–95. 10.1016/j.beproc.2010.11.007 21145379

[23] Range F Aust U Steurer M Huber L. Visual categorization of natural stimuli by domestic dogs. Anim Cogn. (2008) 11:339–47. 10.1007/s10071-007-0123-2 18026761

[24] Fiset S Beaulieu C Landry F. Duration of dogs'(Canis familiaris) working memory in search for disappearing objects. Anim Cogn. (2003) 6:1–10. 10.1007/s10071-002-0157-4 12658530

[25] Kaminski J Call J Fischer J. Word learning in a domestic dog: evidence for “fast mapping”. Science (2004) 304:1682–3. 10.1126/science.1097859 15192233

[26] Hare B Tomasello M. Human-like social skills in dogs? Trends Cogn Sci. (2005) 9:439–44. 10.1016/j.tics.2005.07.003 16061417

[27] Miklósi Á Dog Behaviour, Evolution, and Cognition. Oxford: OUP Oxford (2008).

[28] MacLean EL Herrmann E Suchindran S Hare B Individual differences in cooperative communicative skills are more similar between dogs and humans than chimpanzees. Anim Behav. (2017) 126:41–51. 10.1016/j.anbehav.2017.01.005

[29] Stewart L MacLean EL Ivy D Woods V Cohen E Rodriguez K. Citizen science as a new tool in dog cognition research. PLoS ONE (2015) 10:e0135176. 10.1371/journal.pone.0135176 26376443 PMC4574109

[30] Box GE Cox DR An analysis of transformations. J R Stat Soc Series B (1964) 26:211–52.

[31] Kuhn M Wing J Weston S Williams A Keefer C Engelhardt A caret: Classification and Regression Training. R Package Version 6.0–21. Vienna: CRAN, (2015).

[32] R Core Team R: A Language and Environment for Statistical Computing. Vienna: R Foundation for Statistical Computing (2017).

[33] Kuhn M Johnson K Applied Predictive Modeling, Vol. 26 New York, NY: Springer (2013). 10.1007/978-1-4614-6849-3

[34] MacLean EL Matthews L Hare B Nunn C Anderson R Aureli F. How does cognition evolve? Phylogen Comp Psychol Anim Cogn. (2012) 15:223–38. 10.1007/s10071-011-0448-8 21927850 PMC3980718

[35] Tomasello M Call J Primate Cognition. New York, NY: Oxford University Press (1997).

[36] Hare B. Can competitive paradigms increase the validity of experiments on primate social cognition? Anim Cogn. (2001) 4:269–80. 10.1007/s100710100084 24777517

[37] Bray EE MacLean EL Hare BA Increasing arousal enhances inhibitory control in calm but not excitable dogs. Anim Cogn. (2015) 18:1317–29. 10.1007/s10071-015-0901-1 26169659 PMC4609265

[38] Hare B Plyusnina I Ignacio N Schepina O Stepika A Wrangham R. Social cognitive evolution in captive foxes is a correlated by-product of experimental domestication. Curr Biol. (2005) 15:226–30. 10.1016/j.cub.2005.01.040 15694305

