# Supplementary material for: Enhanced Selection of Assistance and Explosive Detection Dogs Using Cognitive Measures
Source: Front Vet Sci. 2018 Oct 4;5:236. doi: 10.3389/fvets.2018.00236 (PMC6180148; doi:10.3389/fvets.2018.00236)
Supplement: Supplementary file 1 [file Data_Sheet_1.docx]

Supplemental Materials

Procedures for tasks in the Dog Cognition Test Battery

### Familiarization and Warm-ups

#### Reward Preference Warm-ups

This warm-up served to introduce dogs to the rewards used throughout the battery, the choice procedure, and to document dogs’ preferences for the food vs. toy reward.

The task began with two trials to familiarize the dog with the rewards to be presented in test trials. In these familiarization trials, E1 placed a piece of food (trial 1) or a toy (trial 2) on the floor 2m directly in front of the dog. E2 then released the dog’s leash and the dog was allowed to approach the object. Dogs were required to touch the reward on both familiarization trials to advance to the test trials.

In the following six trials, E1 placed a piece of food and the toy in different locations 2m apart from one another and 2m in front of the dog. E2 then released the dog’s leash and the dog was allowed to make a choice. The trial ended when a dog chose either of the objects (i.e. dogs could not choose both objects on the same trial).

#### Hiding-Finding Warm-ups

These trials were conducted each day to familiarize dogs with the object choice paradigm and to assure that subjects were motivated to search for the reward used in the tests. Warm-ups were conducted immediately before the first object choice task on each day of the battery. The warm-up trials consisted of four phases of increasing difficulty.

**Phase 1 – visible reward:** E1 showed the dog the reward and placed it on the floor 2m directly in front of the dog. Dogs were then allowed to approach and obtain the reward. If the dog did not choose the reward, the trial was repeated. Dogs were required to approach the reward on two trials in a row. They had to meet this criterion within a maximum of six trials to advance to Phase 2 warm-ups.

**Phase 2 – one-container, visible placement:** E1 showed the dog the reward and placed it under a container 2m directly in front of the dog. If the dog did not approach and touch the container within 15s, the trial was repeated. Dogs were required to approach and touch the container on two trials (within a maximum of six trials) to advance to Phase 3 warm-ups.

**Phase 3 – two-container, visible placement:** Two containers were placed 2m apart and 2m in front of the dog. E1 showed the dog the reward, and then approached both containers (always beginning at the container to E1’s right), visibly baiting one container and momentarily standing behind the other before returning to the E1 start location. E2 then released the dog to make a choice. If the dog chose the baited container she was allowed to have the reward and the next trial was administered. If the dog chose the un-baited container, E1 revealed that it was empty, the dog was not rewarded, and the trial was repeated until the dog chose correctly. Repeating these trials served to prevent the development of side biases and ensured that dogs gained experience finding the reward in both locations. If a dog did not choose either container within 15s, the trial was repeated. Dogs were required to choose correctly on their first attempt in four out of five consecutive trials to advance to Phase 4 warm-ups.

**Phase 4 – two-container, invisible displacement:** Two containers were placed 2m apart and 2m in front of the dog. E1 showed the dog the reward and then hid it behind a small handheld occluder that E1 carried with her for the remainder of the trial. E1 then walked to only one of the containers and placed the reward under this container, occluding the placement of the reward from the dog’s view, before returning to the E1 start location (dogs were required to infer that E1 had invisibly displaced the reward under the container that E1 approached). E2 then released the dog to make a choice. The reward contingencies, procedure for repeating trials, and performance criteria were identical to Phase 3 warm-ups.

**Warm-up abort criteria:** If a *working dog* did not meet the performance criterion for Phase 2 or Phase 3 warm-ups within 20 trials, the session was aborted. Working dogs were allowed a maximum of 50 trials (across all phases of warm-ups) or 20 minutes to complete warm-ups – whichever was reached first (with the exception of the first day on which they were allowed 30 minutes for the warm-up phase if necessary).

Pet dogs had a slightly different criteria because the dog’s owner was volunteering their time to allow for the testing. If a *pet dog* did not meet the performance criterion for Phase 2 or Phase 3 warm-ups within 40 trials, the session was aborted. Pet dogs were allowed a maximum of 75 trials (across all phases of warm-ups) or 45 minutes to complete warm-ups – whichever was reached first (with the exception of the first day on which they were allowed 60 minutes for the warm-up phase if necessary).

Across all warm-up sessions, we calculated the average number of trials required to meet the criterion within each phase of warm-ups (thus lower scores reflected better performance). We then multiplied this value by -1 so that higher values corresponded to better performance, similarly to the other tasks in the battery.

# Battery Tasks

The Dog Cognition Test Battery (DCTB) consisted of 25 experimental tasks (plus an olfactory control task) based on previously published studies of dog cognition. Tasks are described below in the order in which they were conducted.

## 1. Affect Discrimination

This test measured a dog’s preference to approach an unfamiliar human based on positive or negative affective cues from the human’s tone of voice (based on Vas et al., 2005).

E3 positioned the dog on leash at the start line. E1 and E2 (who had not met the dog prior to this test), sat 2m apart and 2m in front of the dog. E1 and E2 looked directly at the dog and alternated vocalizations for a total of 5s, each vocalizing three times. The experimenter to the dog’s left always vocalized first, and the experimenter to the dog’s right always vocalized last. The ‘unfriendly’ experimenter said “Stop” in a sharp, low tone, while the ‘friendly’ experimenter said “Good” in a high-pitched, affiliative tone. After 5 seconds E3 released the dog’s leash and the dog was allowed 15 seconds to approach E1, E2, or neither. A choice was defined as the dog approaching within arm’s reach of E1 or E2 (verified by E1 or E2 reaching out to touch the dog). If the dog chose the ‘friendly’ experimenter this individual praised and petted the dog. If the dog chose the ‘unfriendly’ experimenter, this individual touched the dog to confirm approach within arm’s length, but otherwise ignored the dog. After the dog made a choice, E3 retrieved the dog and brought her back to the start line. After a choice to either E1 or E2, or if the dog did not approach either individual within 15s, the trial ended. Six trials were conducted: E1 played the ‘friendly’ role for the first three trials and E2 played the ‘friendly role’ for the last three trials (E1 and E2 maintained the same physical positions across all trials). On each trial, a choice to the ‘friendly’ experimenter received a score of 1, an approach to the ‘unfriendly’ experimenter received a score of -1, and no choice received a score of 0. The dependent measure of this task was the dog’s average score across the six trials. Thus, positive scores reflect a tendency to approach the experimenter that made a ‘friendly’ vocalization, negative scores reflect a tendency to approach the experimenter that made an ‘unfriendly’ vocalization, and a score of zero indicates no preference.

## 2. Arm Pointing

This task measured a dog’s ability to use an arm pointing gesture to locate a hidden reward (based on Hare, Call, & Tomasello, 1998; Miklósi et al., 1998).

Two containers were positioned 2m apart, and 2m from the dog. After the containers were baited / sham baited, and as the subject watched, E1 said “look” and pointed to the baited container using the arm closest to it, with her index finger extended and her head turned toward the indicated container. E2 then released the dog to make a choice, and E1 maintained the pointing gesture until the dog made a choice or the maximum trial time had elapsed. Six trials were conducted and the dependent measure was the percent of trials that a dog chose the container toward which E1 gestured.

## 3. Visual Discrimination

This task measured a dog’s ability to learn an arbitrary visual discrimination that predicted the reward’s location (based on Head et al. 1998; Range et al., 2008). On all trials the reward was placed underneath the ‘target container’ but the location of this container, and the location of distractors varied between trials.

**Familiarization to Target Container:** The target container (25cm x 10cm yellow cylindrical container) was placed on the floor in one of three locations (A,C’,A’, Figure S1) 2m in front of the dog. E1 showed the dog the reward and placed it under the target container, concealing this process using a small occluder. E2 then released the dog to make a choice. If the dog chose the target container, E1 lifted the container and allowed the dog to obtain the reward. If the dog did not choose the container within 15s, the trial was repeated. The dog was required to obtain the reward in all three possible locations in order to advance to the test trials.

**Test trials**: The target container and two distractors (blue cups, 12cm x 10cm) were placed on the floor in an equidistant array 2m in front of the dog (A,C’,A’, Figure S1). E1 showed the dog the reward, and then hid it behind the small handheld occluder. E1 then baited or sham-baited each container, beginning with the container to her right and moving leftward, before returning to the E1 start line. E2 then released the dog to make a choice. If the dog chose correctly, E1 lifted the container and allowed the dog to obtain the reward. If the dog chose incorrectly, E1 said ‘wrong’ in a flat, monotone voice, lifted the container to show that it was empty, and encouraged the dog to continue searching until she chose correctly. If the dog did not choose the target container within 15s, E1 led the dog to the correct location, encouraged her to touch the target container, and allowed her to obtain the reward. Consequently across repeated trials each subject had equal opportunity to learn which container always contained the hidden food. Nine trials were conducted, and the location of the target container was counterbalanced across the three locations. The dependent measure for this task was the percentage of trials in which dog’s first choice was to the target container.

## 4. Cylinder

This task measured a dog’s ability to inhibit a prepotent motor response to reach directly for a visible reward on the other side of a transparent barrier (based on Bray, MacLean & Hare, 2013; MacLean et al., 2014).

**Familiarization:** A transparent cylinder (31cm x 10cm) covered with opaque fabric was placed on the floor 1m in front of the dog, and E1 sat on the ground behind it. E1 showed the dog the reward with her right hand and placed it inside the cylinder from the right-side opening. The dog was then allowed 15s to approach the container and obtain the reward. On each trial, E1 recorded whether the dog’s first attempt to obtain the reward was through the front (incorrect) or side (correct) of the cylinder. A response was coded as incorrect if the dog made physical contact with the exterior of the cylinder using its muzzle or paw, whereas a response was coded as correct if the dog’s snout crossed the plane at either open side of the cylinder without first bumping into the exterior of the cylinder. Regardless of accuracy, the dog was allowed to obtain the reward on all trials. The dog was required to respond correctly on four out of five consecutive trials prior to advancing to the test trials.

**Test:** The procedure for the test trials was identical to the familiarization trials, except that the opaque cover was removed from the cylinder, making the cylinder transparent. Thus subjects were required to inhibit approaching the visible reward directly (bumping into the cylinder) in favor of a detour reaching response. As in the familiarization trials, E1 recorded the dog’s first response for each trial, and the dog was allowed to obtain the reward regardless of her first response. Ten test trials were conducted. The dependent measure for this task was the percentage of trials that a dog performed the correct detour response across the ten test trials.

## 5. Detour Navigation

This task measured a dog’s ability to take the shortest route around an obstacle (based on Pongracz et al., 2001).

A 36” Top Paw™ Exercise Pen was positioned in an asymmetrical V shape such that the vertex was .5m in front of the dog with the arms of the fence opening away from the dog. The long arm of the fence (2.44m) was twice the length of the short arm (1.22m). Prior to beginning the test, E2 walked the dog around the barrier to assure that the dog was familiar with the spatial layout. E2 walked the dog around the fence beginning with the long arm (start line ➔ outside of long arm ➔ inside of long arm ➔ inside of short arm ➔ outside of short arm ➔ start line). Once the dog was back at the start line, E1 stood inside the V, centered between the two arms of the fence. E1 showed the dog the reward, then turned so that she was facing away from the dog. E1 then called the dog to come to her. The dog was allowed 30s to detour the barrier and retrieve the reward from E1. During the trial, E1 called the dog continuously until the dog had reached her. Following each successful trial, E2 walked the dog back to the start line via the opposite path the dog used to approach E1 in order to equate motor experiences navigating around both sides of the barrier. If the dog did not detour around the barrier within 30s, the next trial was conducted. After three trials, the barrier was repositioned such that the lengths of the arms were reversed, and E1 familiarized the dog with the configuration for a second time by leading her around the apparatus. Three more trials were conducted in the new orientation. On each trial, the dog obtained a score of 2 for approaching E1 via the shorter route, 1 for reaching E1 via the longer route, and 0 if the dog failed to successfully detour around the barrier. The dependent measure for this task was the average score across trials. Thus, higher scores indicate a greater tendency to take the shortest route around the barrier.

## 6. Spatial Perseveration

This task measured a dog’s ability to inhibit a previously successful motor pattern when the environment changed such that this response was no longer effective (based on Osthaus, Marlow & Ducat, 2010).

At the beginning of the test E2 took the dog out of the room while E1 set up the apparatus. A 36” Top Paw™ Exercise Pen was positioned in the room in a straight line across with width of the room such that that one end of the fencing was flush with the wall on the left side of the room, leaving a .7m gap between the fence and the wall on the right side of the room. Once the fence was positioned, E2 brought the dog back into the room and positioned the dog on leash at the start line. E1 stood in the center of the room on the opposite side of the fence from the dog, showed the dog the reward and said “look!”. E1 then crouched behind the fence and continuously called the dog to come to her after E2 released the dog. After the dog detoured around the fence, E1 rewarded the dog and E2 brought the dog back to the start line. If a dog did not solve the problem in the allotted 30s, E2 showed the dog the solution by walking the dog through the gap. Four trials were conducted with the fence’s gap on the right side of the room. After the completion of these four trials, E2 took the dog out of the room and E1 shifted the fence such that the opening between the fence and the wall was now on the left side of the room. E2 then brought the dog back into the room and four trials were conducted with this new configuration. Thus dogs were required to inhibit the previously successful motor path, in favor of a detour around the other side of the fence.

Scoring: A 1m wide section was marked on the room floor to delineate the center, left, and right areas of the room. Dogs began the trial in the center area but were required to cross into the left or right side of the room to detour the fence. On all trials, E1 recorded the first side of the room (left or right) that the dog entered (operationally defined as a dog’s paw stepping across the lines marking the left and right sides of the room). The dependent measure for this task was the percentage of trials that a dog first moved to the correct side of the room when approaching E1 during the test trials.

## 7. Social Referencing

This task measured a dog’s tendency to look at a human’s face when a social game was interrupted (based on Nagasawa et al., 2009, Merola, Prato-Previde & Marshall-Pescini, 2012).

E knelt in front of the dog holding a stuffed animal (KONG® Wubba Friend). E1 played with the toy in front of the dog for 10s in a manner that was engaging but not overly stimulating (dogs were not allowed to touch or bite the toy during this period). After engaging the dog with this toy for 10s, E1 placed the toy on the floor in front of her and gazed at the dog for the next 20s. During this 20s period, E1 recorded the cumulative time that the dog gazed at her face using a silent stopwatch. The dog was not allowed to have the toy prior to the beginning of the task or during the play period, but E1 gave the dog the toy to play with between trials. Four trials were conducted. The dependent measure for this task was the average percentage of time that a dog gazed at E1’s face during the 20s interruptions in the social game.

## 8. Gaze Direction

This task measured a dog’s ability to use the experimenter’s gaze direction to locate a hidden reward (based on Hare, Call, & Tomasello, 1998; Miklósi et al., 1998).

The gaze direction task was identical to the arm pointing task with the exception of the social gesture that E1 used to inform the dog of the reward’s location. After the apparatus was baited, E1 said “look” and then turned her head to look directly at the baited container. This gesture and vocalization was performed twice, after which E1 maintained her head orientation and gaze at the baited container until the dog made a choice. The dependent measure for this task was the percentage of trials in which a dog chose the container toward which E1 directed her gaze.

## 9. Causal Reasoning

This task measured a dog’s ability to use visual and auditory information to infer the location of a reward (based on Brauer et al., 2006).

**Visual Trials**

*Familiarization to Visual Container* – E1 showed the dog the reward inside of a small bucket (17cm x 16cm) and then placed the bucket at one of two locations 2m apart and 2m in front of the dog. E2 then released the dog to make a choice. If the dog approached the bucket, she was allowed to obtain the reward. The dog was required to approach the bucket one time at each of the two locations before moving on to the visual test trials. These trials served to introduce the dog to finding the reward inside the container used in test trials.

*Visual Test Trials* – Two opaque circular cloths (60cm diameter) were laid flat on the floor 2m apart and 2m in front of the dog. E1 showed the dog the reward inside the bucket and then hid it behind a large occluder before beginning the hiding process. At the location to be baited, E1 lifted the cloth above the occluder, placed the bucket on the ground, and then placed the cloth over top of the bucket such that the bucket visibly displaced the cloth when the occluder was removed. At the unbaited location, E1 similarly lifted the cloth above the occluder and simply placed it smoothly back onto the floor. Thus the cloth lay flat on the floor once the occluder was removed. E2 then released the dog to make a choice. Dogs were required to infer where the bucket containing the reward was hidden based on which of the two cloths was visually displaced by the bucket underneath. We conducted four visual test trials.

**Auditory Trials**

*Familiarization to Auditory Container* – For this portion of the task, E1 used an opaque metal container (20cm x 17cm) that could be shaken (test trials only) such that noise was produced if a hard object was inside the container. E1 presented the reward and then placed it inside the container at one of two locations 2m apart and 2m in front of the dog and returned to the E1 start line. Only one container was used in these familiarization trials. E2 then released the dog to make a choice. If the dog chose the container, E1 opened the container and gave the dog the reward. The dog was required to choose the container one time at each of the two locations before moving on to the auditory test trials. These trials served to introduce the dog to finding the reward inside the container used in test trials.

*Auditory Test Trials* – Two containers (identical to those from familiarization trials) were placed on the floor 2m apart and 2m in front of the dog. E1 showed the dog the reward, and then hid it behind the occluder for the remainder of the baiting/sham-baiting process. At the baited container, E1 opened the container, noisily dropped the reward inside, and then closed the container. She then moved the container in front of the occluder and shook it rhythmically for 3s. Because the reward was hidden inside this container, its contents rattled audibly as E1 shook the container. At the unbaited container, E1 performed similar motions but did not place the reward inside the container. She then shook the (empty) container rhythmically for 3s. Because this container was empty it did not produce noise when shaken. E1 then returned to the E1 start line (Figure S1), and E2 released the dog to make a choice. If a dog chose the baited container, E1 opened the container and allowed the dog to have the reward. If a dog chose the unbaited container E1 opened it to show that it was empty. Dogs were required to infer which container held the reward based on the sounds that each container made when shaken. Four auditory test trials were conducted.

The dependent measure for this task was the percentage of trials that dogs chose the baited container across the visual and auditory test trials.

## 10. Unsolvable Task

This task measured a dog’s tendency to persist at an unsolvable task, or to solicit social help in this context (based on Miklósi et al., 2003)

**Familiarization** – E1 showed the dog the reward and placed it inside a transparent plastic container (14cm x 14cm x 10cm) with the container’s lid loosely in place such that the subject could easily dislodge the lid with her snout or paw. E2 then released the dog who was allowed 30s to approach the container and obtain the reward without assistance from either E1 or E2 The dog was required to successfully obtain the reward by dislodging the container’s lid on four familiarization trials prior to advancing to the test trials. If the dog failed to obtain the reward after twelve familiarization trials, the task was aborted.

**Test** **Trials** – As in familiarization trials, E1 showed the dog the reward and placed it inside the transparent container. However, in these trials E1 locked the container’s lid in place, rendering the problem unsolvable. E2 then released the dog, and moved to the back of the room where she faced the wall for the remainder of the trial. E1 remained standing in the center of the room facing the dog. The subject was allowed 60s to work on the problem individually, or to solicit help (E did not provide help during this time regardless of the dog’s behavior). During this period E1 used a silent stopwatch to record the cumulative time that the dog gazed at her face. At the conclusion of each test trial E1 opened the container and allowed the dog to have the reward. Four test trials were conducted.

The dependent measure from this task was the mean duration of time that a dog gazed at E1’s face across test trials.

## 11. Working Memory

This task measured a dog’s ability to recall the spatial location of a hidden reward after varying temporal delays (based on Dore et al., 1996; Fiset, Beaulieu, & Landry, 2003).

Three containers were placed on the floor in one of three locations 2m in front of the dog (A,C’,A’, Figure S1) . E1 showed the dog the reward and then visibly placed it underneath one of the containers. She then positioned the freestanding occluder in front of the containers and E2 started a countdown timer for 20, 40 or 60s. Both E1 and E2 stood silently without looking at or engaging with the dog during the delay. At the conclusion of the delay, E1 removed the occluder and E2 released the dog to make a choice. If the dog did not choose within 15s, the next trial was conducted (trials on which the dog did not respond were not repeated for memory tasks because failure to choose may reflect the subject either forgetting the reward’s location, or forgetting that the reward had been hidden altogether). However, trials on which the dog did not choose were excluded from analysis so that scores reflected the percentage of correct choices out of the total number of choices a subject made. Nine trials were conducted (Trials 1-3 – 20s, Trials 4-6 – 40s, Trials 7-9 – 60s), and the location of the reward was counterbalanced across trials within each delay. If a subject did not choose on at least ⅔ of trials (i.e. 6 or more choices), his or her partial data were excluded from analysis (1% of dogs). The dependent measure for this task was the percentage of trials that a dog chose the baited container.

## 12. Sensory Bias

This task measured whether a dog prioritized visual or olfactory information when these senses were pitted against one another (based on Szetei et al., 2003).

**Familiarization** – Two plastic funnels (19cm base x 19cm tall) were used to hide the rewards in this task. Each funnel was attached to a small wooden stick (31cm x 3cm) such that E1 could tip the funnel back to reveal an item during the visual cue, and E2 could stand on the stick to keep the funnel in place during the olfactory cue. The funnels were positioned with the spout facing upward so that a dog could smell (but not see) the funnel’s contents by sniffing at the opening of the spout.

Two familiarization trials, in which only a single funnel was used, were conducted prior to test trials. These trials served to familiarize the dog with finding food in the containers to be used in test trials. E1 first showed the dog the reward, and then baited the funnel concealing this process from the dog using the occluder. The dog then received both an olfactory and visual cue that the reward was located under the funnel. The olfactory cue consisted of E2 walking the dog to the funnel and allowing the dog to smell the opening at the top of the funnel for 3s. The visual cue consisted of E1 lifting the funnel such that the reward was visible to the dog for 3s (while the dog was standing at the start line). For one familiarization trial, the dog received the visual cue first, and for the second trial, the dog received the olfactory cue first. The dog was required to choose the funnel one time at each of the locations prior to test trials.

**Test Trials** – In test trials, E1 created the illusion that only a single reward was being hidden, while in reality, both funnels were baited on every trial. The funnels were positioned 2m apart and 2m in front of the dog. Before beginning the test trials, while E2 distracted the dog, E1 surreptitiously placed a reward under one of the containers. At the start of each trial, E1 showed the dog the reward and hid it behind the occluder for the remainder of the baiting/sham-baiting process. E1 then visited both containers and (behind the occluder) baited the one that did not already contain a reward such that both containers now contained identical rewards. The dog then received a visual cue that the reward was in one location, and an olfactory cue that it was in the other. The visual and olfactory cues were identical to those from familiarization trials and whether the visual or olfactory cue was presented first was counterbalanced across trials. E2 then released the dog to make a choice, and choices to either funnel were rewarded. Eight test trials were conducted. For the first four test trials, the dog received the visual cue first, and for the last four test trials, the dog received the olfactory cue first. The dependent measure for this task was percentage of trials that a dog chose the visually-cued container. Thus, higher scores indicate a visual bias whereas lower scores indicate an olfactory bias.

## 13. Marker Cue

This task measured a dog’s ability to infer the location of hidden reward when E1 used an arbitrary physical marker to communicatively indicate the reward’s location (based on Agnetta, Hare & Tomasello, 2000; Riedel et al., 2006).

This marker cue task was procedurally identical to the arm pointing task with the exception of the social gesture that E1 used to inform the dog of the reward’s location. After the containers were baited / sham baited, E1 said “look” and showed the dog a small blue wooden block (5cm x 5 cm x 5cm) which she held in her right hand. E1 then stood briefly behind each of the containers and placed the wooden block on top of the baited container. Six trials were conducted and the dependent measure for this task was the percentage of trials that a dog chose the container on which E1 placed the wooden block.

## 14. Odor Discrimination

This task measured a dog’s ability to discriminate and remember which of two containers contained food after smelling the contents of each container (based on Szetei et al., 2003).

Two plastic funnels (identical to those from Sensory Bias) were used as hiding containers. The funnels were positioned with the spout facing upward so that a dog could smell (but not see) the funnel’s contents by sniffing at the opening of the spout.

**Familiarization** –Two familiarization trials were conducted using a single funnel at the left or right choice locations. After E1 baited the container, E2 walked the dog to the container, placed her hand on the funnel to secure it, said “sniff it”, and allowed the dog 3s to smell the funnel’s contents (the phrase “sniff it” was unfamiliar to all dogs tested and was intended only to capture the dogs attention so they would observe E2 as she placed her hand on the funnel). E2 then walked the dog back to the starting position and released the dog to make a choice. Subjects were required to choose the baited funnel one time at the left and right locations before advancing to the test.

**Discrimination Test** – E2 positioned the dog on leash at the start line. The two visually identical funnels were placed 2m apart and 2m in front of the dog. After E1 baited the apparatus, E2 walked the dog to both containers in the order in which they were baited. As in familiarization trials, at each funnel E2 placed her hand on the funnel to secure it, said “sniff it”, and allowed the dog 3s to smell the funnel’s contents. E2 then returned the dog to the start line and released the dog to make a choice. We conducted six odor discrimination trials.

**Masked Odor Test** – The procedure for the masked trials was identical to the unmasked trials, except that the containers used were visually identical funnels with a distracting odor placed inside. The distracting odor was a peach-flavored tea bag (Bigelow Perfect Peach Herbal Tea) taped to the inside of the funnel. Fresh tea bags were used for each subject. Thus dogs were required to smell and remember the location of the reward in the context of a distracting odor that was present at both locations. We conducted six masked odor trials.

If the dog did not make a choice on a total of twelve trials across the entire task, including the familiarization trials, the task was aborted. The dependent measure for this task was the percentage of trials that a dog chose the baited funnel.

## 15. Perspective Taking

This task measured a dog’s tendency to obey or disobey a command depending on whether the experimenter was watching the dog (based on Call et al., 2003).

**Sit Test** – E1 stood 1m in front of the dog either facing towards (facing condition) or away from (back-turned condition) the dog. E2 positioned the dog in a standing position at the start line (Figure S1, location H). Once the dog was in position, E1 said the dog’s name, followed by a sit command. As soon as E1 gave the command, E2 released the dog’s leash and started a timer for 10s. E2 recorded whether or not the dog sat or lay down during the following 10s.

E rewarded the dog (with food) regardless of performance, but no verbal praise was given if the dog did not sit or lie down. Four trials were conducted, two in the facing condition and two in the back-turned condition. The overall score for the sit test was the percentage of trials that a dog sat in the facing condition minus the percentage of trials that a dog sat in the back-turned condition. Thus, positive scores reflect a greater tendency to obey E1’s command when she was watching than when she was not.

**Stay Test** – E2 positioned the dog on leash at the start line. E1 showed the dog the reward and told the dog to sit. Once the dog was sitting, E1 placed the reward 1m in front of the dog and held her hand upright above the reward, palm facing the dog, and said ‘stay’ using stern tone of voice. E1 then stepped back 1m from the reward, and either remained facing the dog (facing condition) or turned her back to the dog (back-turned condition). Once E1 was in position, E2 released the dog’s leash and started a 30s timer. If the dog approached the prohibited item, she was allowed to obtain it but was not praised. If the dog did not take the reward with 30s, E1 praised the dog, picked up the reward and gave it to the dog. We conducted four trials, two with E1 facing the dog, and two with E1’s back turned. The overall score for the stay test was the percentage of trials that a dog took the prohibited reward when E1’s back was turned minus the percentage of trials that a dog took the prohibited reward when E1 was watching. Thus higher scores reflect a greater tendency to disobey E1’s command when she was not watching than when she was.

The dependent measure for this task was a composite metric averaging the score from the sit test and the stay test. Higher scores reflect a greater tendency to obey the commands when E1 was watching compared to when she was not.

## 16. Spatial Transpositions

This task measured a dog’s ability to track the location of baited container over the course of a variety of spatial transpositions (based on Rooijakkers, Kaminski & Call, 2009).

E sat on the floor 2m in front of the dog. Two containers were positioned on the floor 2m in front of the dog and in the positions shown in Figure S2. E1 visibly baited one container and then changed the container positions according to the spatial transpositions shown in Figure S2. At the conclusion of each spatial transposition, both containers were equidistant from the dog. We conducted three types of spatial transpositions. In ‘no cross’ trials, both containers shifted .25 m to the right or left. In ‘one cross’ trials, one container crossed in front of the other (stationary) container ending up at the opposite end of the array. In ‘two cross’ trials, both containers crossed paths, each ending at the other container’s starting location. We conducted a total of 12 trials, consisting of blocks of 4 ‘no cross trials’, followed by 4 ‘one-cross’ trials, followed by 4 ‘two-cross’ trials. The dependent measure for this task was the percentage of correct responses across trials.

## 17. Transparent Obstacle

This task measured a dog’s ability to inhibit approaching E1 directly when E1 called the dog urgently from the opposite side of a transparent barrier (based on Bray, MacLean & Hare, 2013).

**Apparatus & Familiarization:** A V-shaped transparent barrier was positioned in the center of the room. Each of the two side panels was 80cm wide and 1m tall and the vertex of the apparatus consisted of a flat transparent 40cm panel, behind which the experimenter stood. During the trials, E1 kneeled on a wooden block (60cm x 60cm x 10cm) positioned inside the V-shaped barrier. Prior to starting the task, E2 walked the dog around the perimeter of the apparatus to familiarize the dog with the spatial arrangement, and the path required to detour around the obstacle to obtain the reward from E1.

**Test Trials:** Standing on the opposite side of the transparent barrier from the dog, E1 showed the dog the reward and began to call the dog to come to her. E1 called the dog in an urgent and high-pitched tone of voice encouraging the dog to come to her as quickly as possible. E2 released the dog three seconds after E1 began to call the dog, and E1 continued to call the dog until the dog had detoured the obstacle to reach her. Because the barrier was transparent, the dog had to resist the impulse to rush toward E1 directly (bumping into the barrier) in favor of a detour around the barrier. On each trial we recorded whether the dog attempted to approach E1 through the barrier (incorrect; operationally defined as making physical contact with the exterior of the barrier using the forepaw or muzzle), or whether the dog successfully navigated around the barrier (correct) without first bumping into it. Subjects were allowed a total of 120s to successfully navigate around the barrier on each trial. If the dog did not initiate a response within 20s of E1 calling the dog, the trial was repeated. We conducted 5 trials and the dependent measure for this task was the percentage of trials that a dog made the correct response.

## 18.Memory - Distraction

This task measured a dog’s ability to remember the location of a hidden reward following varying temporal delays during which E2 intentionally distracted the dog (based on Dore et al., 1996; Fiset, Beaulieu, & Landry, 2003).

The general method was identical to the working memory task with the exceptions that during the delay E2 stood between the dog and the occluded containers and pet the dog’s head while singing to distract the dog. At the conclusion of the delay E1 removed the occluder in front of the containers, E2 moved to a position beside the dog, and released the dog to make a choice. We conducted six trials. The first three trials imposed a 20s delay/distraction period, and the final three trials imposed a 40s delay/distraction period. Similarly to the working memory task, if a subject did not choose on ⅔ of trials (i.e., 4 or more choices) his or her partial data were excluded from analysis (1% of dogs). The dependent measure for this task was the percentage of trials that a dog chose the baited container.

## 19. Contagious Yawning

This task measured whether a dog yawned contagiously when hearing an audio recording of an unfamiliar human yawning (based on Silva, Bessa, & de Sousa, 2012; Joly-Mascheroni, Senju, & Shepherd, 2008; Harr, Gilbert & Phillips, 2009; O’Hara & Reeve, 2011; ).

Auditory stimuli were obtained from Silva et. al (2012) because these stimuli were shown to induce contagious yawning in a previous study with dogs. The stimuli consisted of an audio recording of a human yawning, and a control stimulus consisting of the yawning clip played backwards. The stimuli were presented in an ABBA design (A = yawning, B = control). Each phase of the design was 75s long during which the auditory stimulus was played as a continuous loop. Stimuli were played through Logitech S-120 Portable Speakers (5cm x 17cm) located centrally in the testing room. During the test subjects were allowed to move freely throughout the room. E1 remained outside the room throughout the test, and E2 sat quietly in the corner of the room using a handheld camera to record the dog’s behavior (E2 wore a bandana over her mouth and nose to conceal her face from the dog). Videos were subsequently coded for the total number of yawns (defined as a mouth gape with the corners of the lip retracted) during the yawning and control conditions. The dependent measure for this task was the number of yawns a dog made during the yawning condition minus the number of yawns in the control condition. Thus higher scores reflect a greater tendency to yawn during the yawning than the control stimuli.

## 20. Reaching

This task measured a dog’s ability to use a reaching gesture to infer the location of a hidden reward (based on Bräuer et al, 2006).

The reaching task was procedurally identical to the arm pointing task with the exception of the social gesture that E1 used to inform the dog of the reward’s location. After the containers were baited / sham baited, E1 sat in a chair equidistant between the two containers, looked at the subject, said ‘look!’, and reached toward the baited container. E1 performed the reaching gesture three times, each time extending her arm fully and grasping effortfully toward the baited container. After the third reaching movement E1 placed her hands on her thighs, looked downward, and E2 released the dog to make a choice. We conducted six trials and the dependent measure for this task was the percentage of trials in which dogs chose the container toward which E1 reached.

## 21. Inferential Reasoning

This task measured a dog’s ability to infer the location of a hidden reward through the principle of exclusion (based on Erdohegyi et al., 2007).

Two containers were positioned 2m apart, and 2m in front of the dog. After the containers were baited / sham baited, E1 walked to each container performing the designated action at each container. At the baited container, E1 simply gripped the baited container with her right hand for 1s. At the un-baited container, E1 grasped the container and lifted it for 1s to reveal to the subject that no reward was hidden underneath. E1 then returned to her starting position, looked downward, and E2 released the dog to make a choice. Dogs were required to infer the location of the reward through knowledge that it was *not* in one of the two possible hiding locations. We conducted six trials and the dependent measure for this task was the percentage of trials that a dog chose the baited container.

## 22. Odor Control Trials

This task served to verify that subjects could not locate the hidden reward using olfaction (based on Miklósi et al., 1998; Brauer et al., 2006; Hare et al., 2002).

The procedure was identical to the arm pointing task with the exception that after baiting / sham baiting the containers, E1 did not provide a social cue, and instead simply looked downward. E2 then released the dog to make a choice. We conducted six trials and the dependent measure for this task was the percentage of trials that a dog chose the baited container.

## 23. Laterality (First Step)

This task measured which forelimb (left or right) a dog preferred to use when initiating a step off of a platform, as well as the consistency of this preference across trials (based on Tomkins, Thomson & McGreevy, 2010).

A 10cm tall wooden platform (60cm x 60cm) was used as a step and placed in the center of the room. Before beginning, E2 coaxed the dog onto and off of the step a few times to familiarize the dog with the apparatus. Each trial began by E2 positioning the dog in a sit position on the platform with the dog facing toward E1. The dog was positioned as close as possible to the front of the platform. Once the dog was sitting in position, E1 stood 1m directly in front of the dog and called the dog to her. On each trial we coded which paw (left or right) the dog led with (the first paw to touch the ground) as the dog stepped off the platform to approach E1. After each trial E1 gave the dog a piece of food and E2 walked the dog back to the starting position on the platform. We conducted a total of 26 trials. For the first 13 trials, E2 stood with the dog on her left side, and for the second 13 trials, E2 stood with the dog on her right side. If the task could not be completed within 15 minutes, or if the dog could not be coaxed onto the platform within the first 5 minutes, the task was aborted. The dependent measure for this task was the percentage of trials that a dog led with her right foot.

## 24. Laterality (Object Manipulation)

This task measured which forepaw (left or right) a dog preferred to use when physically manipulating an object, as well as the consistency of this preference across trials (based on Batt, Batt & McGreevy, 2007; Branson & Rogers, 2006).

The apparatus used for this task was a 30cm long piece of 2.5cm diameter PVC pipe. Approximately 5cm of each end of the tube were filled with a soft food reward, such as peanut butter or Kong Stuff’n. The tube was placed on the floor, and the dog was allowed to manipulate it freely with her paws and/or mouth. From video we coded which paw the dog used to manipulate the tube (hereafter a touch) as the dog stabilized the tube to consume the food within. A touch was defined as one of the paws making physical contact with the tube. If a dog touched the tube with both paws simultaneously, this event was not coded. If the dog stabilized the tube for more than 3s, E2 gently pulled the tube away from the dog and repositioned it in ~.5m front of the dog with the opening facing the dog in order not to bias which paw the dog used for subsequent touches.

This task was completed after a dog had accumulated a total of 26 touches (similarly to the first step measure), or fifteen minutes had elapsed. In cases where 15 minutes elapsed before the dog had performed 26 touches, all touches performed within the time limit were retained for analysis. The task was aborted if a dog did not perform a touch within 5 minutes of beginning the task.

## 25. Rotation

This task measured whether a dog encoded the spatial location of a hidden reward using egocentric or allocentric spatial cues (based on Fiset, Gagnon, & Beaulieu, 2000).

**Familiarization** – Two containers were placed 2m apart and 1m in front of the dog. E1 showed the dog the reward and then visibly placed it in the container to her right (the dog’s left). E2 then released the dog to make a choice. If the dog chose correctly, E1 removed the container and allowed the dog to obtain the reward before E2 led the dog back to the E2 start location. If the dog chose incorrectly, E1 said ‘wrong’ in a flat tone of voice, showed the dog that this container was empty, and allowed the dog to continue searching until she either chose the correct container or 15s had elapsed. If the dog did not choose the baited container within 15s, the trial was repeated. This procedure continued until the dog had located the reward in the container to E1’s right a total of five times.

**Test Trials** – In test trials, the dog was positioned at the opposite side of the room from familiarization trials, reversing the dog’s visual perspective of the two containers. Similarly, E1 now stood at the opposite side of the room behind the two containers. While E2 distracted the dog, E1 surreptitiously placed a reward in one container at this time. E1 then showed the dog the reward, hid it behind an occluder and proceeded to bait or sham bait each of the two containers (E placed the reward under the container that was not already baited so that a reward was hidden under both containers at the conclusion of the baiting process). After E1 returned to a position equidistant between the containers, E2 released the dog to make a choice. In this task, choosing either container was rewarded, and the container that was not chosen remained baited for the following trial (during which E1 would also bait the other container). We conducted five test trials.

The dependent measure for this task was the percentage of trials that a dog choose the reward to her left during test trials (the same location that was baited during familiarization trials, but to the opposite side of the dog in test trials). Thus, higher scores reflect an allocentric search bias whereas lower scores reflect an egocentric search bias.

## 26. Retrieval

This task measured a dog’s tendency to return a retrieved object to in front of the experimenter (based on Hare, Call, & Tomasello, 1998; Gacsi et al., 2004; Horn et al., 2012).

**Warm-up –**Prior to beginning the test, E1 attempted to establish a game of fetch with the dog. E1 showed the dog a tennis ball, and then threw it across the room encouraging the dog to bring it back to her to throw again. After this 1 minute warm-up, test trials began.

**Test –** E1 stood at a central location in the room and tossed the ball toward one end of the room. Depending on the condition, E1 either remained facing the direction in which she threw the ball, or turned her back. Subjects were then allowed 15s to retrieve the ball and bring it back to E1. On each trial, we coded the dog’s behavior according to the following scoring system: 0 – the dog did not retrieve the object; 1 – the dog retrieved the object but did not bring it back to within arm’s reach of E; 2 – the dog retrieved the object but brought it to E1’s back (i.e. returned to location where E1 could not see the dog); 3 – the dog retrieved the object and brought it in front of E1 (i.e. returned to a location where E1 could see the dog). Scores of two or three required that the dog returned to within arm’s reach of E1, standing either behind, or in front of E1, respectively. We conducted 4 trials. For two trials, E1 remained facing the direction in which she threw the ball, and for the other two trials E1 turned her back after throwing the ball. The dependent measure for this task was the average score across trials.

**References**

Agnetta, B., Hare, B., & Tomasello, M. (2000). Cues to food location that domestic dogs (*Canis familiaris*) of different ages do and do not use. *Animal Cognition, 3*(2), 107-112.

Andreeff, M., Ruvolo, V., Gadgil, S., Zeng, C., Coombes, K., Chen, W., et al. (2008). HOX expression patterns identify a common signature for favorable AML. *Leukemia, 22*(11), 2041-2047.

Batt, L., Batt, M., & McGreevy, P. (2007). Two tests for motor laterality in dogs. *Journal of Veterinary Behavior-Clinical Applications and Research, 2*(2), 47-51.

Branson, N. J., & Rogers, L. J. (2006). Relationship between paw preference strength and noise phobia in Canis familiaris. *Journal of Comparative Psychology, 120*(3), 176-183.

Brauer, J., Kaminski, J., Riedel, J., Call, J., & Tomasello, M. (2006). Making inferences about the location of hidden food: Social dog, causal ape. *Journal of Comparative Psychology, 120*(1), 38-47.

Bray, E. E., MacLean, E. L., & Hare, B. A. (2013). Context specificity of inhibitory control in dogs. *Animal Cognition*, 1-17.

Budaev, S. (1998). How many dimensions are needed to describe temperament in animals: A factor reanalysis of two data sets. *International Journal of Comparative Psychology, 11*, 17-29.

Call, J., Brauer, J., Kaminski, J., & Tomasello, M. (2003). Domestic dogs (*Canis familiaris*) are sensitive to the attentional state of humans. *Journal of Comparative Psychology, 117*(3), 257-263.

Carpenter, M., Nagell, K., & Tomasello, M. (1998). Social cognition, joint attention, and communicative competence from 9 to 15 months of age. *Monographs of the Society for Research in Child Development, 63*(4), 1-143.

Carpenter, M., Pennington, B. F., & Rogers, S. J. (2002). Interrelations among social-cognitive skills in young children with autism. *Journal of autism and developmental disorders, 32*(2), 91-106.

Cieri, R. L., Churchill, S. E., Franciscus, R. G., Tan, J., & Hare, B. (2014). Craniofacial feminization, social tolerance, and the origins of behavioral modernity. *Current Anthropology, 55*(4), 419-443.

de Waal, F. B. M., & Tyack, P. L. (2003). *Animal social complexity : intelligence, culture, and individualized societies*. Cambridge, Mass.: Harvard University Press.

Dore, F. Y., Fiset, S., Goulet, S., Dumas, M. C., & Gagnon, S. (1996). Search behavior in cats and dogs: Interspecific differences in working memory and spatial cognition. *Learning & behavior, 24*(2), 142-149.

Erdohegyi, A., Topal, J., Viranyi, Z., & Miklosi, A. (2007). Dog-logic: inferential reasoning in a two-way choice task and its restricted use. *Animal Behaviour, 74*, 725-737.

Fiset, S., Beaulieu, C., & Landry, F. (2003). Duration of dogs'(Canis familiaris) working memory in search for disappearing objects. *Animal Cognition, 6*(1), 1-10.

Fiset, S., Gagnon, S., & Beaulieu, C. (2000). Spatial encoding of hidden objects in dogs (*Canis familiaris*). *Journal of Comparative Psychology, 114*(4), 315.

Gácsi, M., Györi, B., Gyoöri, B., Virányi, Z., Kubinyi, E., Range, F., et al. (2009). Explaining dog wolf differences in utilizing human pointing gestures: selection for synergistic shifts in the development of some social skills. *PloS One, 4*(8), e6584.

Gacsi, M., Miklosi, A., Varga, O., Topal, J., & Csanyi, V. (2004). Are readers of our face readers of our minds? Dogs (*Canis familiaris*) show situation-dependent recognition of human's attention. *Animal Cognition, 7*(3), 144-153.

Goodall, J., Lonsdorf, E. V., Ross, S. R., & Matsuzawa, T. (2010). *The mind of the chimpanzee: ecological and experimental perspectives*: University of Chicago Press.

Hair, J. F., Anderson, R. E., Tatham, R. L., & Black, W. (1998). Multivariate data analysis, 1998. *Upper Saddle River*.

Hare, B., Brown, M., Williamson, C., & Tomasello, M. (2002). The domestication of social cognition in dogs. *Science, 298*(5598), 1634-1636.

Hare, B., Call, J., & Tomasello, M. (1998). Communication of food location between human and dog (*Canis familiaris*). *Evolution of Communication, 2*(1), 137-159.

Hare, B., Plyusnina, I., Ignacio, N., Schepina, O., Stepika, A., Wrangham, R., et al. (2005). Social cognitive evolution in captive foxes is a correlated by-product of experimental domestication. *Current Biology, 15*(3), 226-230.

Hare, B., & Tomasello, M. (2005). Human-like social skills in dogs? *Trends in Cognitive Sciences, 9*(9), 439-444.

Hare, B., Wobber, V., & Wrangham, R. (2012). The self-domestication hypothesis: evolution of bonobo psychology is due to selection against aggression. *Animal Behaviour, 83*(3), 573-585.

Hare, B., & Woods, V. (2013). *The Genius of Dogs: How Dogs Are Smarter than You Think*: Dutton Adult.

Harr, A. L., Gilbert, V. R., & Phillips, K. A. (2009). Do dogs (Canis familiaris) show contagious yawning? *Animal Cognition, 12*(6), 833-837.

Head, E., Callahan, H., Muggenburg, B., Cotman, C., & Milgram, N. (1998). Visual-discrimination learning ability and β-amyloid accumulation in the dog. *Neurobiology of aging, 19*(5), 415-425.

Herrmann, E., Call, J., Hernandez-Lloreda, M. V., Hare, B., & Tomasello, M. (2007). Humans have evolved specialized skills of social cognition: the cultural intelligence hypothesis. *Science, 317*(5843), 1360-1366.

Herrmann, E., Hare, B., Call, J., & Tomasello, M. (2010). Differences in the Cognitive Skills of Bonobos and Chimpanzees. *Plos One, 5*(8).

Herrmann, E., Hernandez-Lloreda, M. V., Call, J., Hare, B., & Tomasello, M. (2010). The Structure of Individual Differences in the Cognitive Abilities of Children and Chimpanzees. *Psychological Science, 21*(1), 102-110.

Horn, L., Virányi, Z., Miklósi, A., Huber, L., & Range, F. (2012). Domestic dogs (*Canis familiaris*) flexibly adjust their human-directed behavior to the actions of their human partners in a problem situation. *Animal cognition, 15*, 57-71.

Joly-Mascheroni, R. M., Senju, A., & Shepherd, A. J. (2008). Dogs catch human yawns. *Biology Letters, 4*(5), 446-448.

Kaiser, H. F. (1970). A second generation little jiffy. *Psychometrika, 35*(4), 401-415.

Kaiser, H. F., & Rice, J. (1974). Little Jiffy, Mark IV. *Educational and psychological measurement*.

Kaminski, J., Call, J., & Fischer, J. (2004). Word learning in a domestic dog: evidence for "fast mapping". *Science, 304*(5677), 1682-1683.

Kaminski, J., & Nitzschner, M. (2013). Do dogs get the point? A review of dog–human communication ability. *Learning and Motivation, 44*(4), 294-302.

Kerr, M. K., & Churchill, G. A. (2001). Bootstrapping cluster analysis: assessing the reliability of conclusions from microarray experiments. *Proceedings of the National Academy of Sciences, 98*(16), 8961-8965.

MacLean, E. L., Hare, B., Nunn, C. L., Addessi, E., Amici, F., Anderson, R. C., et al. (In Press). The Evolution of Self-Control. *Proceedings of the National Academy of Sciences*.

MD Anderson Cancer Center. OOMPA: Object-Oriented Microarray and Proteomic Analysis.

Merola, I., Prato-Previde, E., & Marshall-Pescini, S. (2012). Social referencing in dog-owner dyads? *Animal cognition, 15*, 175-185.

Miklosi, A., Kubinyi, E., Topal, J., Gacsi, M., Viranyi, Z., & Csanyi, V. (2003). A simple reason for a big difference: Wolves do not look back at humans, but dogs do. *Current Biology, 13*(9), 763-766.

Miklósi, Á., Polgárdi, R., Topál, J., & Csányi, V. (1998). Use of experimenter-given cues in dogs. *Animal Cognition, 1*(2), 113-121.

Nagasawa, M., Kikusui, T., Onaka, T., & Ohta, M. (2009). Dog's gaze at its owner increases owner's urinary oxytocin during social interaction. *Hormones and Behavior, 55*(3), 434-441.

O'Hara, S. J., & Reeve, A. V. (2011). A test of the yawning contagion and emotional connectedness hypothesis in dogs, *Canis familiaris*. *Animal Behaviour, 81*(1), 335-340.

Osthaus, B., Marlow, D., & Ducat, P. (2010). Minding the gap: spatial perseveration error in dogs. *Animal Cognition, 13*(6), 881-885.

Pilley, J. W., & Reid, A. K. (2011). Border collie comprehends object names as verbal referents. *Behavioural Processes, 86*(2), 184-195.

Plomin, R. (2001). The genetics of g in human and mouse. *Nature Reviews Neuroscience, 2*(2), 136-141.

Pongracz, P., Miklosi, A., Kubinyi, E., Gurobi, K., Topal, J., & Csanyi, V. (2001). Social learning in dogs: the effect of a human demonstrator on the performance of dogs (*Canis familiaris*) in a detour task. *Animal Behaviour, 62*(6), 1109-1117.

R Core Team. (2015). R: A Language and Environment for Statistical Computing. Vienna, Austria: R Foundation for Statistical Computing.

Range, F., Aust, U., Steurer, M., & Huber, L. (2008). Visual categorization of natural stimuli by domestic dogs. *Animal cognition, 11*(2), 339-347.

Revelle, W. (2014). psych: Procedures for personality and psychological research. *Northwestern University, Evanston. R package version, 1*(1).

Riedel, J., Buttelmann, D., Call, J., & Tomasello, M. (2006). Domestic dogs (*Canis familiaris*) use a physical marker to locate hidden food. *Animal Cognition, 9*(1), 27-35.

Rooijakkers, E., Kaminski, J., & Call, J. (2009). Comparing dogs and great apes in their ability to visually track object transpositions. *Animal cognition, 12*(6), 789-796.

Russell, J. L., Lyn, H., Schaeffer, J. A., & Hopkins, W. D. (2011). The role of socio‐communicative rearing environments in the development of social and physical cognition in apes. *Developmental science, 14*(6), 1459-1470.

Silva, K., Bessa, J., & de Sousa, L. (2012). Auditory contagious yawning in domestic dogs (Canis familiaris): first evidence for social modulation. *Animal Cognition*, 1-4.

Stevens, J. P. (2012). *Applied multivariate statistics for the social sciences*. New York: Routledge.

Sutter, N. B., & Ostrander, E. A. (2004). Dog star rising: the canine genetic system. *Nature Reviews Genetics, 5*(12), 900-910.

Szetei, V., Miklosi, A., Topal, J., & Csany, V. (2003). When dogs seem to lose their nose: an investigation on the use of visual and olfactory cues in communicative context between dog and owner. *Applied Animal Behaviour Science, 83*(2), 141-152.

Téglás, E., Gergely, A., Kupán, K., Miklósi, Á., & Topál, J. (2012). Dogs' gaze following is tuned to human communicative signals. *Current Biology, 22*(3), 209-212.

Tomasello, M. (1999). *The cultural origins of human cognition*. Cambridge, Mass.: Harvard University Press.

Tomasello, M., & Call, J. (2008). Assessing the Validity of Ape-Human Comparisons: A Reply to Boesch (2007). *Journal of Comparative Psychology, 122*(4), 449-452.

Tomkins, L. M., Thomson, P. C., & McGreevy, P. D. (2010). First-stepping Test as a measure of motor laterality in dogs (*Canis familiaris*). *Journal of Veterinary Behavior-Clinical Applications and Research, 5*(5), 247-255.

Topal, J., Gergely, G., Erdohegyi, A., Csibra, G., & Miklosi, A. (2009). Differential Sensitivity to Human Communication in Dogs, Wolves, and Human Infants. *Science, 325*(5945), 1269-1272.

Udell, M. A. R., Dorey, N. R., & Wynne, C. D. L. (2010). What did domestication do to dogs? A new account of dogs' sensitivity to human actions. *Biological Reviews of the Cambridge Philosophical Society, 85*, 327-345.

Vas, J., Topal, J., Gacsi, M., Miklosi, A., & Csanyi, V. (2005). A friend or an enemy? Dogs' reaction to an unfamiliar person showing behavioural cues of threat and friendliness at different times. *Applied Animal Behaviour Science, 94*(1-2), 99-115.

Virányi, Z., Gácsi, M., Kubinyi, E., Topál, J., Belényi, B., Ujfalussy, D., et al. (2008). Comprehension of human pointing gestures in young human-reared wolves (*Canis lupus*) and dogs (*Canis familiaris*). *Animal Cognition, 11*(3), 373-387.

Wobber, V., Herrmann, E., Hare, B., Wrangham, R., & Tomasello, M. (2013). Differences in the early cognitive development of children and great apes. *Developmental psychobiology, 56*(3), 547-573.
